# Supplementary material for: Molecular mechanisms of exceptional lifespan increase of Drosophila melanogaster with different genotypes after combinations of pro-longevity interventions
Source: Commun Biol. 2022 Jun 9;5:566. doi: 10.1038/s42003-022-03524-4 (PMC9184560; doi:10.1038/s42003-022-03524-4)
Supplement: Supplementary file 2 — Supplementary Information [file 42003_2022_3524_MOESM2_ESM.pdf]

Molecular mechanisms of exceptional lifespan increase of *Drosophila melanogaster* with different genotypes after combinations of pro-longevity interventions

Mikhail V. Shaposhnikov, Zulfiya G. Guvatova, Nadezhda V. Zemskaya, Liubov A. Koval, Eugenia V. Schegoleva, Anastasia A. Gorbunova, Denis A. Golubev, Natalya R. Pakshina, Natalia S. Ulyasheva, Ilya A. Solovev, Margarita A. Bobrovskikh, Nataly E. Gruntenko, Petr N. Menshanov, George S. Krasnov, Anna V. Kudryavseva, Alexey A. Moskalev

Supplementary Information

Supplementary Figures

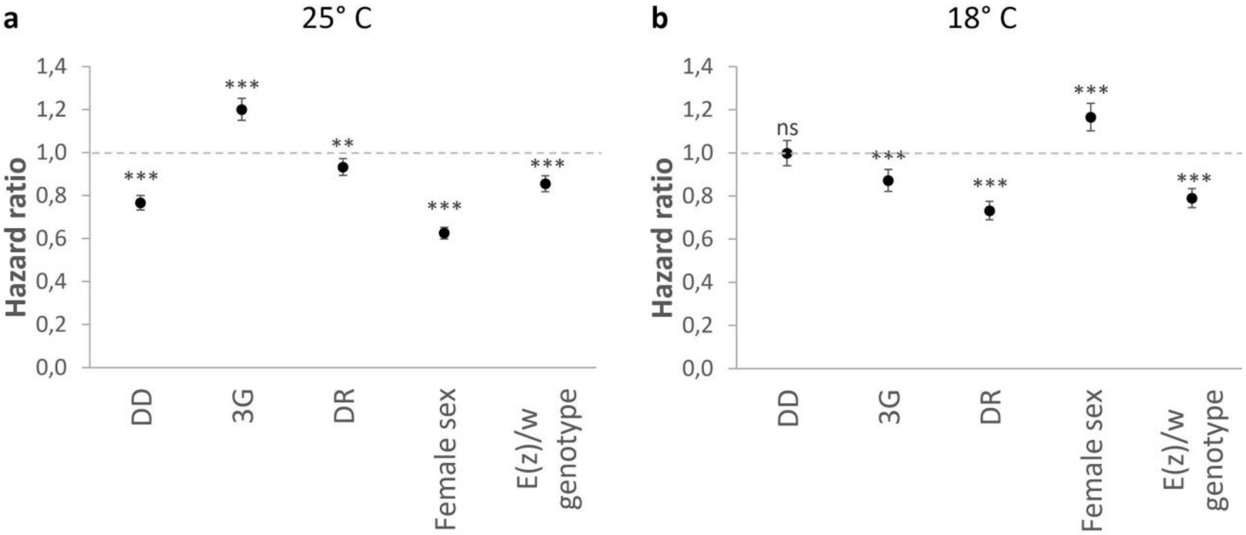

Supplementary Figure 1. Cox regression analysis was performed separately for control and experimental temperature variants. a, 25°C, b, 18°C. Dashed lines indicate a hazard ratio of 1, which corresponds to reference conditions (12 h light : 12 h dark, no substances added, normal diet, male sex, and *w/w* genotype). Error bars designate 95% confidence intervals. Asterisks (*) indicate the level of statistical significance of differences (*p<0.05; **p<0.01; ***p<0.001); ns – not significant. Bonferroni correction was used in all multiple comparisons.

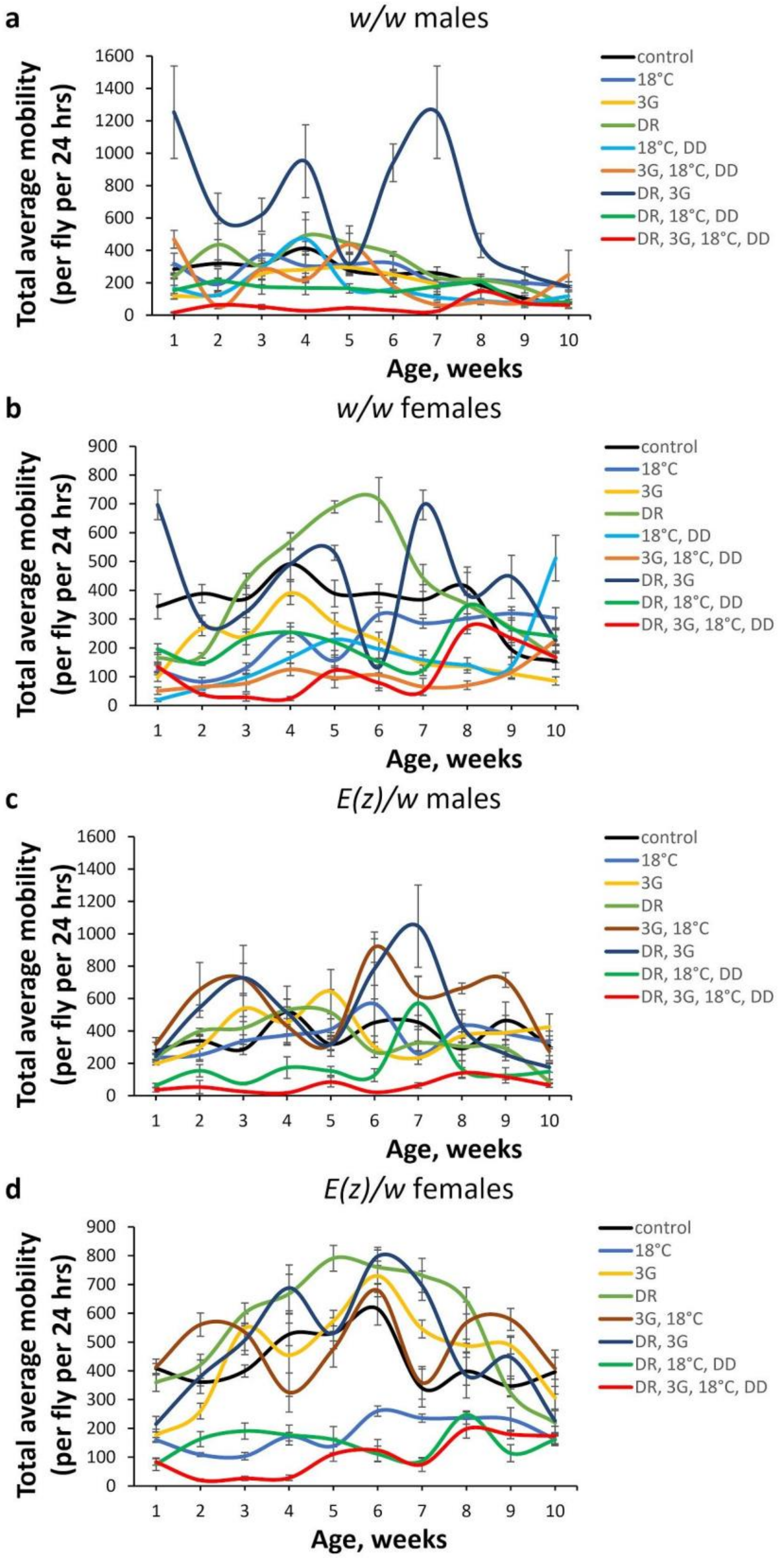

Supplementary Figure 2. Locomotor activity under different combinations of experimental conditions. The error bars show standard errors. See Supplementary Tables 4–7 for the data analyses.

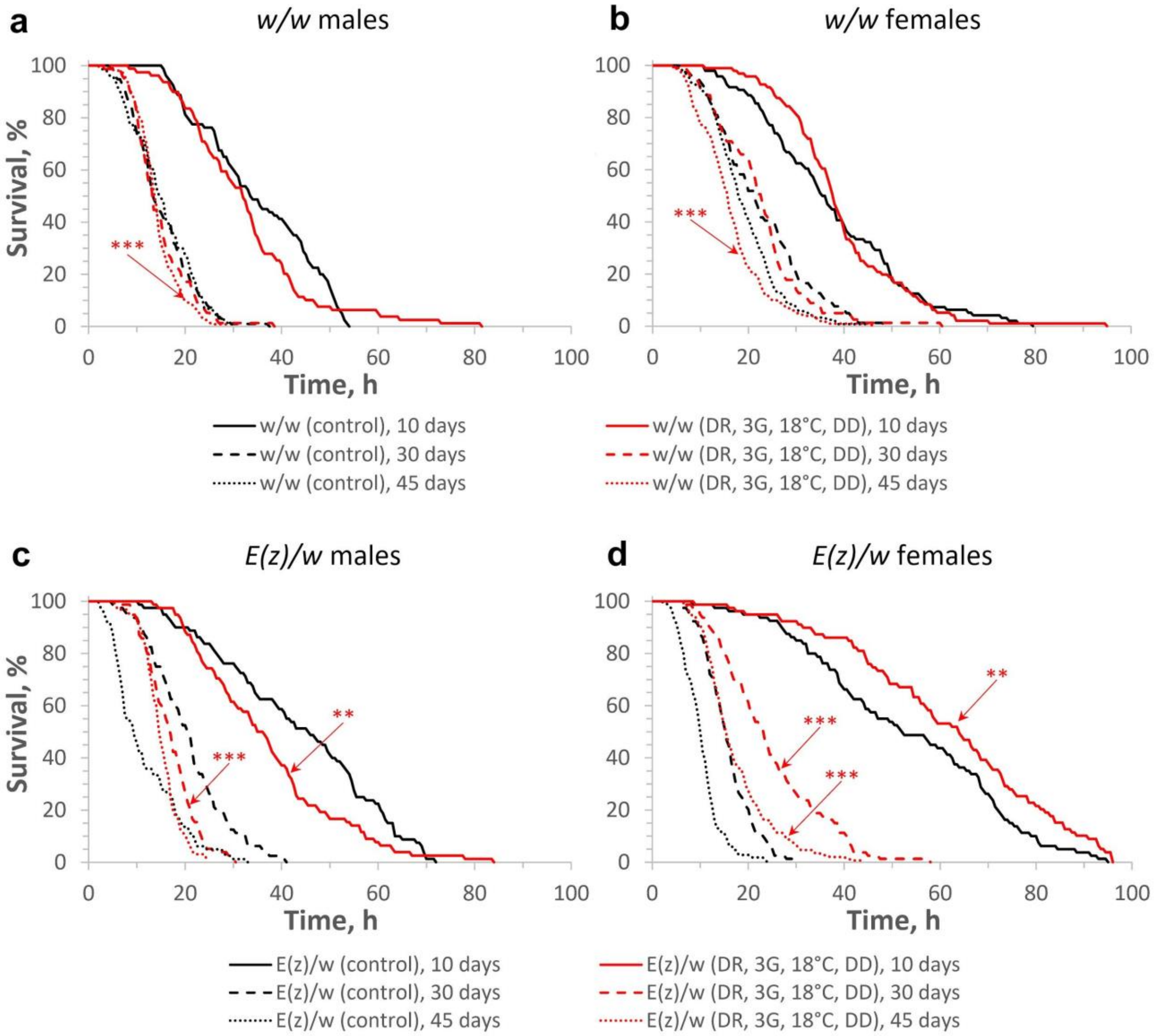

Supplementary Figure 3. Effects of a combination of experimental conditions (dietary restriction (DR), geroprotectors (3G), low ambient temperature (18°C), and maintenance in the dark (DD)) on age-dependent changes in the resistance to oxidative stress induced by 20 mM paraquat. a, c, Males. b, d, Females. a, b, Control line *w/w*. c, d, Long-lived line *E(z)/w*. Asterisks (*) indicate the level of statistical significance of differences (*p<0.05; **p<0.01; ***p<0.001, log-rank test with Bonferroni corrections). See Supplementary Tables 4–7 for the data analyses.

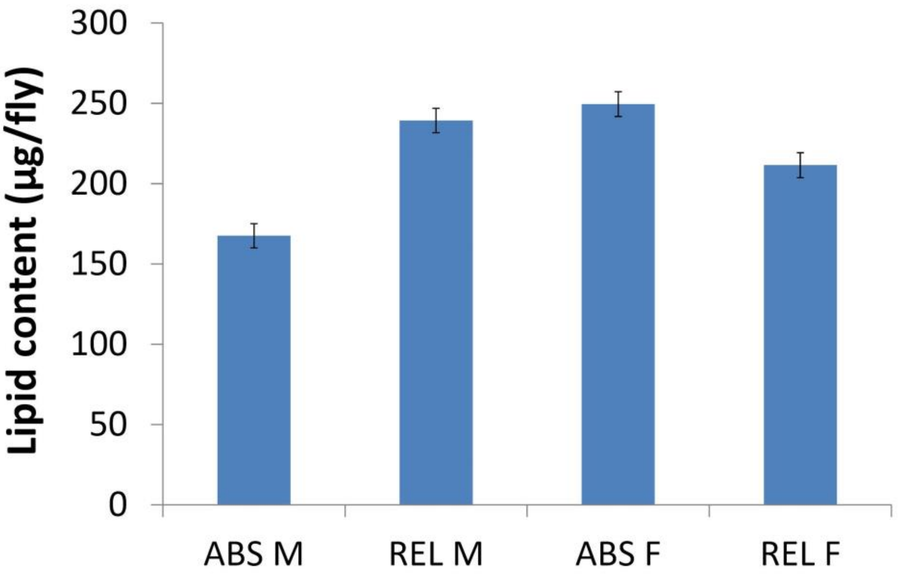

Supplementary Figure 4. The absolute and relative lipid levels in *D. melanogaster* males (ABS M and REL M) and females (ABS F and REL F). Each value is an average of 8-12 measurements. Means±SEM are indicated.

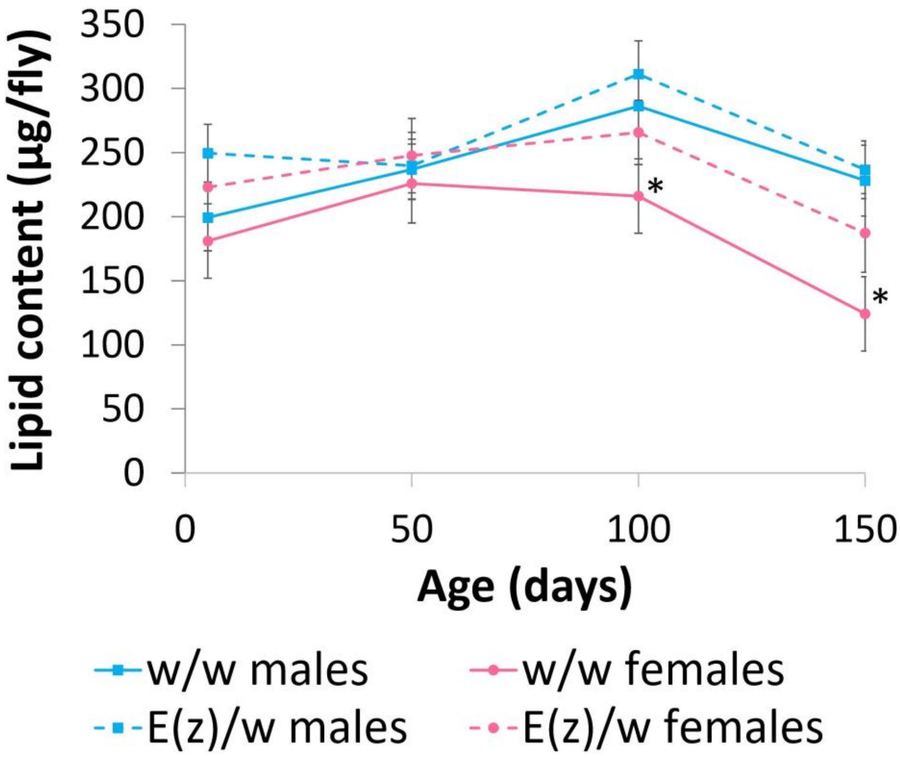

Supplementary Figure 5. Comparison of total lipid content in different sexes of *E(z)/w* and *w/w* lines exposed to the combined action of dietary restriction, co-administration of berberine, fucoxanthin, and rapamycin, constant darkness, and low temperature (18°C) conditions. Each data point represents a mean of 8-12 biological replicates ±SEM. *$p<0.05$ differences between the age-matched adult flies of *E(z)/w* and *w/w* lines.

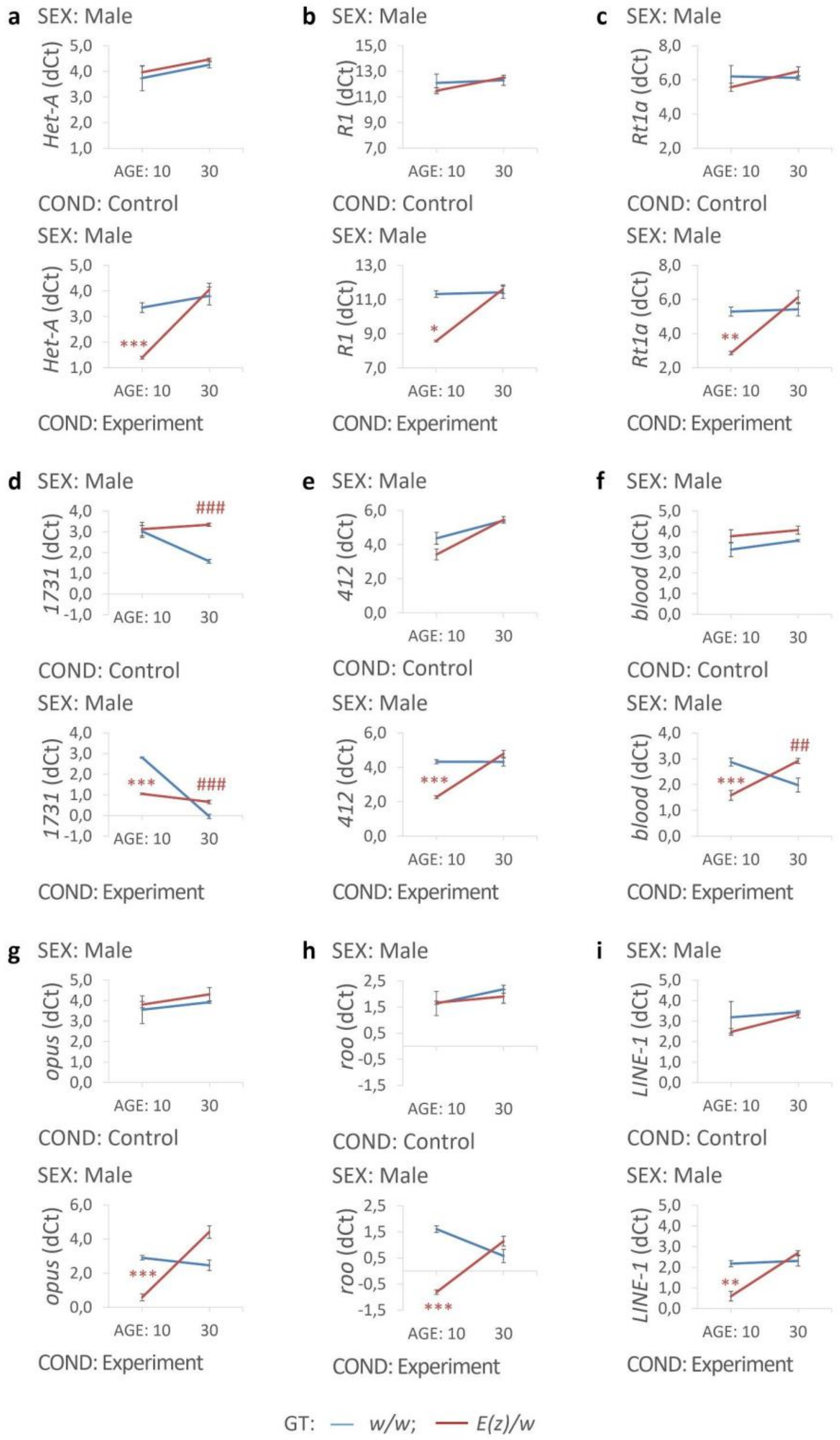

Supplementary Figure 6. Effects of conditions (COND), age (AGE), and genotype (GT) on the activity of 9 retrotransposon families (*Het-A1* (a), *R1* (b), *Rt1α* (c), *1731* (d), *412* (e), *blood* (f), *opus* (g), *roo* (h), and *LINE-1* (i)) in male flies**.** Asterisk (*) indicates the level of statistical significance of differences between flies maintained in the experimental (COND: Experiment) and control (COND: Control) conditions (*p<0.05; **p<0.01; ***p<0.001). Hash (#) indicate the level of statistical significance of differences between *E(z)/w* and *w/w* flies ($^{\#}$p<0.05; $^{\#\#}$p<0.01; $^{\#\#\#}$p<0.001). Three-way Bayesian ANCOVA followed by post-hoc Bonferroni test was used to determine statistical significance. The error bars show standard errors, ns – not significant.

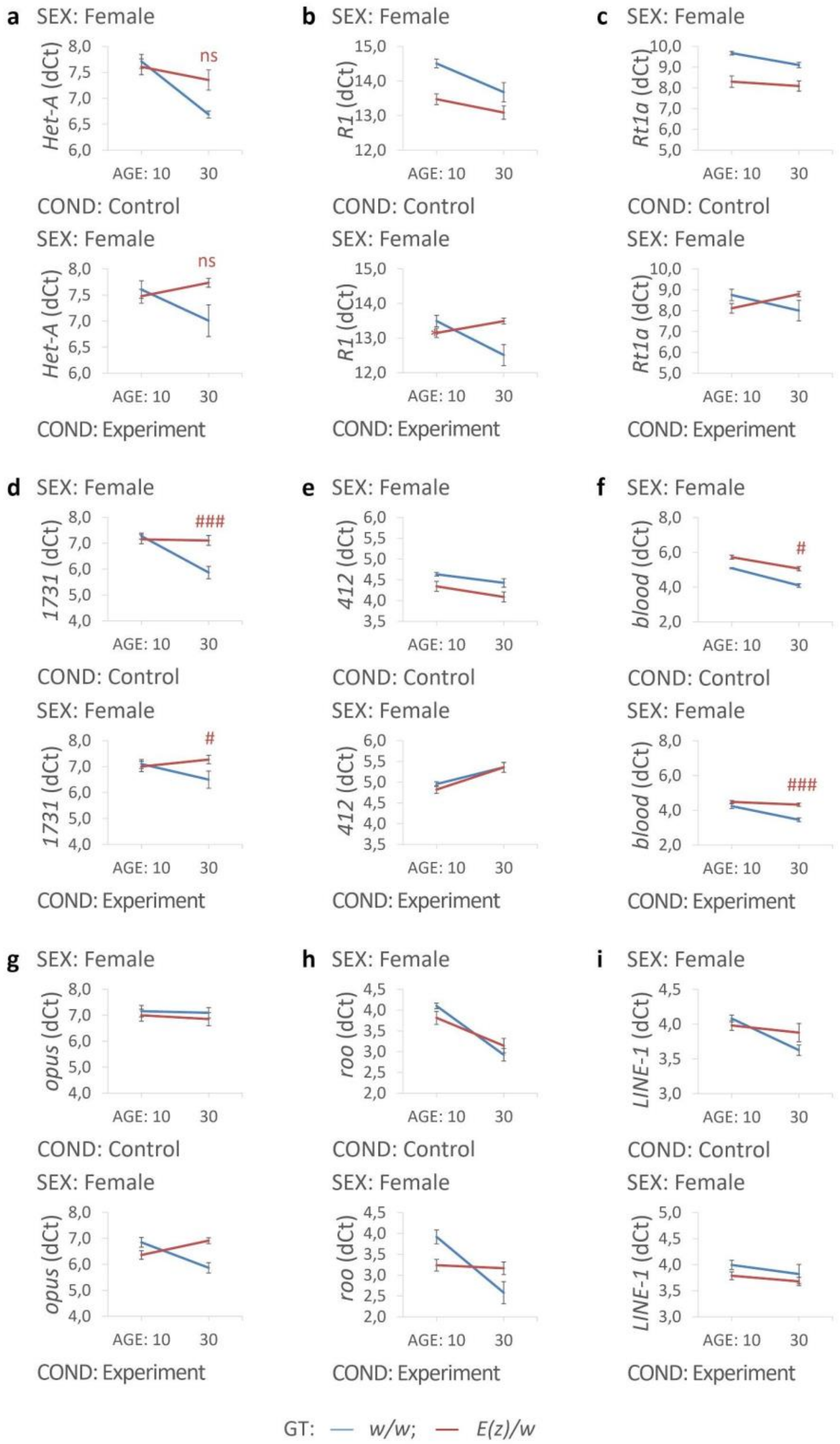

Supplementary Figure 7. Effects of conditions (COND), age (AGE), and genotype (GT) on the activity of 9 retrotransposon families (*Het-A1* (a), *R1* (b), *Rt1α* (c), *1731* (d), *412* (e), *blood* (f), *opus* (g), *roo* (h), and *LINE-1* (i)) in female flies**.** Asterisk (*) indicates the level of statistical significance of differences between flies maintained in the experimental (COND: Experiment) and control (COND: Control) conditions (*p<0.05; **p<0.01; ***p<0.001). Hash (#) indicate the level of statistical significance of differences between *E(z)/w* and *w/w* flies ($^{\#}$p<0.05; $^{\#\#}$p<0.01; $^{\#\#\#}$p<0.001). Three-way Bayesian ANCOVA followed by post-hoc Bonferroni test was used to determine statistical significance. The error bars show standard errors, ns – not significant.

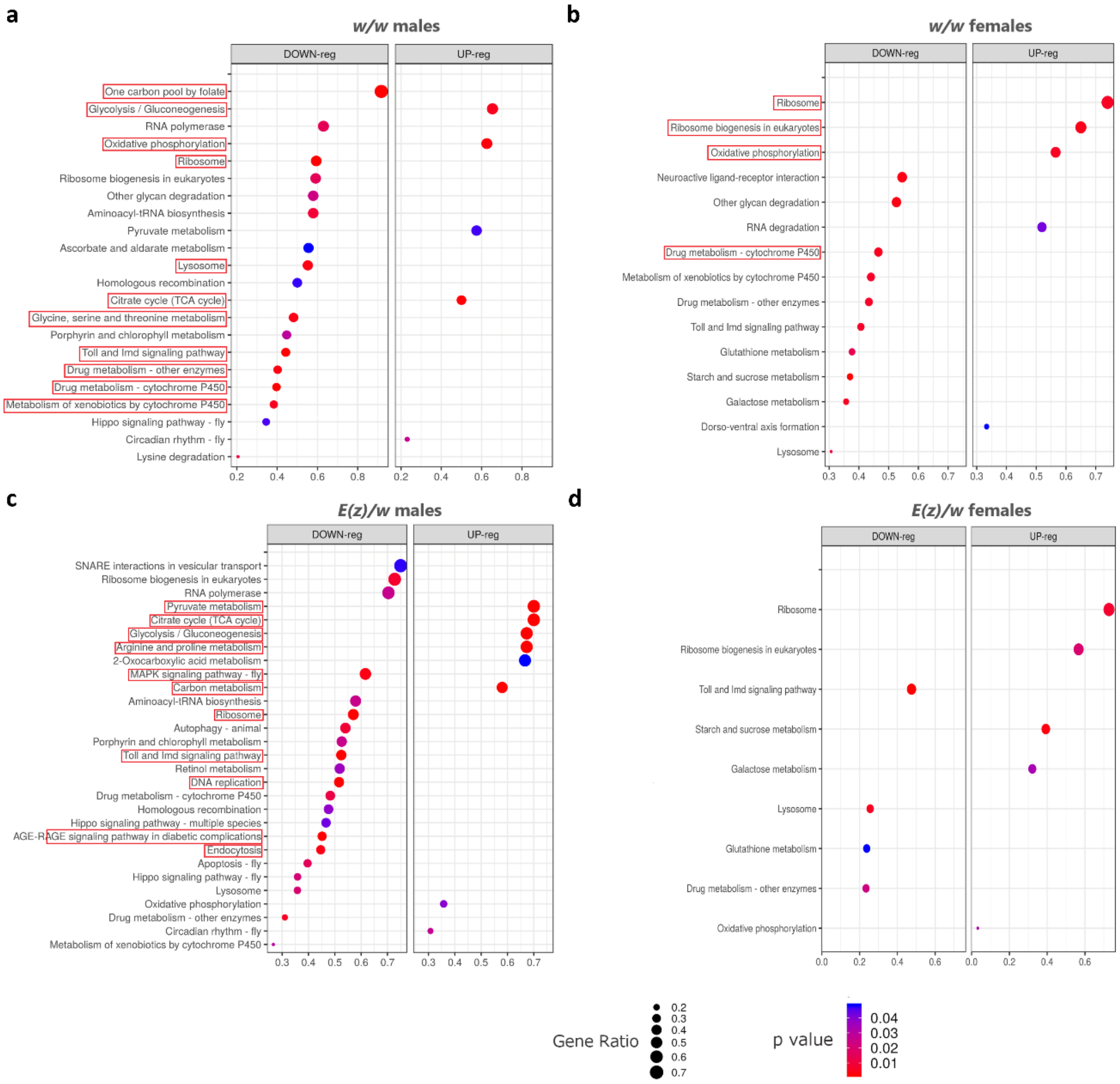

Supplementary Figure 8. Dotplots showing the results of KEGG pathways enrichment analyses performed for DE genes (either up-regulated or down-regulated) associated with exposure to combination of anti-aging interventions (dietary restriction (DR); co-administration of berberine, fucoxanthin, and rapamycin (3G); living in constant darkness (DD) and low temperature (18°C) conditions) in 50-day-old *D. melanogaster*. (a) *w/w* males, (b) *w/w* females, (c) *E(z)/w* males, (d) *E(z)/w* females. The x-axis and dot size indicate gene ratio (the number of DE genes involved in the KEGG pathway divided by total number of genes that are annotated as participants of this pathway). Dot color indicates the enrichment test FDR (false discovery rate) according to Fisher's exact test.

Supplementary Tables

Supplementary Table 1. The effects of different factor combinations on lifespan parameters of *w/w* control and *E(z)/w* long-lived strains

Variant	Sex	M (days)	dM (%)	p (M)	90% (days)	d90% (%)	p (90%)	Max (days)	dMax (%)	n
w/w (control)	♂	58	n/a	n/a	80	n/a	n/a	90	n/a	366
w/w (DD)	♂	57	-2	8.2E+00	85	+6	**1.5E-03**	93	+3	134
w/w (18°C)	♂	112	+93	**2.4E-11**	143	+79	**1.8E-11**	162	+80	310
w/w (3G)	♂	59	+2	3.3E+00	79	-1	7.9E+00	90	0	245
w/w (DR)	♂	59	+2	3.8E+00	73	-9	**7.2E-09**	86	-4	318
w/w (18°C, DD)	♂	113	+95	**2.6E-11**	155	+94	**2.3E-11**	170	+89	237
w/w (3G, DD)	♂	61	+5	2.7E+00	85	+6	**2.7E-01**	93	+3	238
w/w (3G, 18°C)	♂	147	+153	**2.0E-11**	162	+103	**3.5E-12**	173	+92	134
w/w (DR, DD)	♂	76	+31	**1.2E-11**	83	+4	**1.7E-07**	87	-3	148
w/w (DR, 18°C)	♂	146	+152	**2.6E-11**	164	+105	**1.8E-11**	178	+98	399
w/w (DR, 3G)	♂	76	+31	**2.3E-11**	87	+9	**9.0E-10**	91	+1	139
w/w (3G, 18°C, DD)	♂	130	+124	**1.7E-11**	162	+103	**1.1E-11**	182	+102	386
w/w (DR, 18°C, DD)	♂	146	+152	**2.4E-11**	167	+109	**9.8E-12**	181	+101	145
w/w (DR, 3G, DD)	♂	61	+5	8.4E+00	82	+3	**3.0E-02**	90	0	146
w/w (DR, 3G, 18°C)	♂	152	+162	**3.0E-11**	167	+109	**5.6E-12**	181	+101	142
w/w (DR, 3G, 18°C, DD)	♂	153	+164	**3.3E-11**	171	+114	**6.9E-12**	184	+104	376
w/w (control)	♀	66	n/a	n/a	79	n/a	n/a	86	n/a	370
w/w (DD)	♀	72	+9	**2.1E-04**	81	+3	5.1E-02	85	-1	152
w/w (18°C)	♀	113	+71	**1.8E-11**	134	+70	**6.8E-12**	144	+67	131
w/w (3G)	♀	70	+6	**1.4E-02**	84	+6	**1.1E-10**	90	+5	343
w/w (DR)	♀	66	0	4.9E+00	74	-6	**2.0E-06**	83	-3	338
w/w (18°C, DD)	♀	91	+38	**1.1E-08**	142	+80	**2.7E-11**	163	+90	213
w/w (3G, DD)	♀	65	-2	1.3E+01	75	-5	6.6E-02	82	-5	262
w/w (3G, 18°C)	♀	116	+76	**1.0E-11**	145	+84	**1.5E-11**	155	+80	108
w/w (DR, DD)	♀	76	+15	**2.4E-08**	83	+5	**5.1E-08**	90	+5	159
w/w (DR, 18°C)	♀	124	+88	**2.1E-11**	146	+85	**9.5E-12**	166	+93	293
w/w (DR, 3G)	♀	76	+15	**1.8E-11**	84	+6	**7.1E-12**	90	+5	151
w/w (3G, 18°C, DD)	♀	121	+83	**3.3E-11**	147	+86	**3.6E-12**	168	+95	357

w/w (DR, 18°C, DD)	♀	126	+91	**1.3E-11**	154	+95	**9.8E-12**	164	+91	147
w/w (DR, 3G, DD)	♀	76	+15	**1.7E-07**	87	+10	**9.0E-12**	90	+5	128
w/w (DR, 3G, 18°C)	♀	121	+83	**1.3E-11**	152	+92	**4.7E-12**	162	+88	155
w/w (DR, 3G, 18°C, DD)	♀	149	+126	**1.7E-11**	164	+108	**8.9E-12**	185	+115	366
E(z)/w (control)	♂	77	n/a	n/a	88	n/a	n/a	95	n/a	242
E(z)/w (DD)	♂	63	-18	**2.5E-11**	80	-9	**1.2E-06**	94	-1	333
E(z)/w (18°C)	♂	165	+114	**1.5E-11**	175	+99	**6.6E-12**	194	+104	135
E(z)/w (3G)	♂	49	-36	**2.7E-11**	65	-26	**1.3E-11**	92	-3	567
E(z)/w (DR)	♂	62	-19	**2.7E-11**	79	-10	**1.4E-08**	98	+3	668
E(z)/w (18°C, DD)	♂	121	+57	**8.3E-06**	155	76	**3.4E-12**	164	+73	90
E(z)/w (3G, DD)	♂	68	-12	**1.2E-11**	79	-10	**5.0E-04**	93	-2	238
E(z)/w (3G, 18°C)	♂	154	+100	**1.2E-11**	175	+99	**6.4E-13**	183	+93	85
E(z)/w (DR, DD)	♂	69	-10	**2.4E-11**	79	-10	**1.4E-03**	100	+5	150
E(z)/w (DR, 18°C)	♂	157	+104	**8.8E-12**	182	+107	**5.0E-13**	197	+107	171
E(z)/w (DR, 3G)	♂	55	-29	**2.1E-11**	72	-18	**9.2E-13**	90	-5	314
E(z)/w (3G, 18°C, DD)	♂	106	+38	**2.7E-11**	143	+63	**9.1E-13**	164	+73	127
E(z)/w (DR, 18°C, DD)	♂	155	+101	**1.1E-11**	178	+102	**2.8E-12**	191	+101	139
E(z)/w (DR, 3G, DD)	♂	74	-4	4.5E-01	88	0	7.8E+00	102	+7	137
E(z)/w (DR, 3G, 18°C, DD)	♂	171	+122	**2.2E-11**	187	+113	**2.8E-11**	213	+124	333
E(z)/w (control)	♀	88	n/a	n/a	98	n/a	n/a	105	n/a	221
E(z)/w (DD)	♀	76	-14	**2.8E-11**	87	-11	**1.1E-12**	103	-2	347
E(z)/w (18°C)	♀	140	+59	**1.7E-11**	167	+70	**5.2E-13**	174	+66	158
E(z)/w (3G)	♀	62	-30	**3.4E-11**	83	-15	**3.4E-12**	94	-10	646
E(z)/w (DR)	♀	71	-19	**3.1E-11**	88	-10	**9.2E-12**	99	-6	676
E(z)/w (18°C, DD)	♀	106	+20	**1.7E-02**	158	+61	**1.2E-12**	164	+56	143
E(z)/w (3G, DD)	♀	77	-13	**2.4E-11**	89	-9	**2.8E-07**	100	-5	267
E(z)/w (3G, 18°C)	♀	133	+51	**1.8E-05**	161	+64	**1.8E-12**	172	+64	77
E(z)/w (DR, DD)	♀	78	-11	**2.0E-11**	92	-6	**9.7E-08**	95	-10	141
E(z)/w (DR, 18°C)	♀	151	+72	**2.2E-11**	178	+82	**1.4E-11**	196	+87	160
E(z)/w (DR, 3G)	♀	68	-23	**1.7E-11**	86	-12	**1.1E-11**	94	-10	290
E(z)/w (3G, 18°C, DD)	♀	85	-3	1.3E+01	149	+52	**2.8E-12**	164	+56	128
E(z)/w (DR, 18°C, DD)	♀	149	+69	**1.1E-11**	183	+87	**4.9E-13**	191	+82	132

E(z)/w (DR, 3G, DD)	♀	85	-3	**8.0E-02**	96	-2	1.4E+01	99	-6	154
E(z)/w (DR, 3G, 18°C, DD)	♀	165	+88	**2.0E-11**	187	+91	**6.7E-12**	200	+90	333

Maintaining in the dark (DD), low ambient temperature (18°C), combination of rapamycin, berberine and fucoxanthin (3G), dietary restriction (DR). Sex: males (♂) and females (♀); M (days) - median lifespan; 90% (days) - age of 90% mortality; Max (days) - age of 100% mortality; dM (%), d90% (%), - differences between median lifespan, age of 90% mortality and age of 100% mortality of control and experimental flies, respectively; n - number of flies; p(M) and p(90%) – Bonferroni corrected p-value for Fisher's exact test to determine the differences in median lifespan and age of 90% mortality, respectively; n/a – not applicable. Significant differences ($p<0.05$) in bold.

Supplementary Table 2. Cox proportional hazards analysis of different conditions influencing the lifespan of *w/w* and *E(z)/w* flies

Genotype	Sex	Risk factor	Hazard ratio	Hazard ratio 95% lower	Hazard ratio 95% upper	p-value^{BC}
w/w	♂	DD	0.89220	0.81578	0.97577	0.05010
w/w	♂	18°C	0.28270	0.25354	0.31522	**0.00000**
w/w	♂	3G	0.89175	0.81600	0.97454	**0.04569**
w/w	♂	DR	0.90713	0.83013	0.99127	0.12502
w/w	♀	DD	0.84707	0.78807	0.91048	**0.00003**
w/w	♀	18°C	0.09047	0.08018	0.10207	**0.00000**
w/w	♀	3G	0.65722	0.61387	0.70364	**0.00000**
w/w	♀	DR	0.60225	0.56214	0.64523	**0.00000**
E(z)/w	♂	DD	0.69835	0.64968	0.75066	**0.00000**
E(z)/w	♂	18°C	0.03645	0.03054	0.04349	**0.00000**
E(z)/w	♂	3G	1.48397	1.38301	1.59229	**0.00000**
E(z)/w	♂	DR	0.81682	0.76401	0.87328	**0.00000**
E(z)/w	♀	DD	0.75672	0.70569	0.81144	**0.00000**
E(z)/w	♀	18°C	0.09430	0.08276	0.10745	**0.00000**
E(z)/w	♀	3G	1.21742	1.13904	1.30119	**0.00000**
E(z)/w	♀	DR	0.78130	0.73077	0.83531	**0.00000**

Sex: males (♂) and females (♀); Risk factor: maintaining in the dark (DD), low temperature (+18°C), combination of rapamycin, berberine and fucoxanthin (3G), dietary restriction (DR). p-value^{BC} – Bonferroni corrected p value. Significant differences ($p \leq 0.05$) in bold.

Supplementary Table 3. Cox proportional hazards analysis of conditions, sex, and genotype influencing the Supplementary lifespan at different ambient temperature

Ambient temperature	Risk factors	Hazard ratio	Hazard ratio 95% lower	Hazard ratio 95% upper	p-value^{BC}
25°C	DD	0.76539	0.73252	0.79974	**0.00000**
25°C	3G	1.19929	1.14960	1.25113	**0.00000**
25°C	DR	0.93190	0.89392	0.97149	**0.00446**
25°C	Female sex	0.62470	0.59880	0.65171	**0.00000**
25°C	*E(z)/w* genotype	0.85444	0.81810	0.89239	**0.00000**
18°C	DD	0.99764	0.94081	1.05791	1.00000
18°C	3G	0.87119	0.82214	0.92316	**0.00002**
18°C	DR	0.73115	0.68965	0.77514	**0.00000**
18°C	Female sex	1.16428	1.10259	1.22941	**0.00000**
18°C	*E(z)/w* genotype	0.78880	0.74553	0.83459	**0.00000**

Risk factor: maintaining in the dark (DD), combination of rapamycin, berberine and fucoxanthin (3G), dietary restriction (DR), sex (female), genotype (*E(z)/w*). p-value^{BC} – Bonferroni corrected p value. Significant differences ($p<0.05$) in bold.

SupplementaryTable 4. Analysis of differences in locomotor activity factored by age and genotype using two-way ANOVA

Source of variation	SS	DF	MS	F	p-value
w/w males					
Intercept	261517510	1	261517510	1111.355	0.000000
Age	21171606	9	2352401	9.997	0.000000
Treatment	114211076	1	114211076	485.356	0.000000
Age×Variant	35511310	9	3945701	16.768	0.000000
Error	22590161	96	235314	n/a	n/a
w/w females					
Intercept	438338934	1	438338934	905.4046	0.000000
Age	19793995	9	2199333	4.5428	0.000084
Treatment	94765825	1	94765825	195.7422	0.000000
Age×Variant	71870656	9	7985628	16.4946	0.000000
Error	36794335	76	484136	n/a	n/a
E(z)/w males					
Intercept	541693694	1	541693694	519.9144	0.000000
Age	36632341	9	4070260	3.9066	0.000410
Treatment	296428445	1	296428445	284.5102	0.000000
Age×Variant	32080824	9	3564536	3.4212	0.001402
Error	79183657	76	1041890	n/a	n/a
E(z)/w females					
Intercept	635009706	1	635009706	1258.866	0.000000
Age	36676870	9	4075208	8.079	0.000000
Treatment	233250581	1	233250581	462.404	0.000000
Age×Variant	61301521	9	6811280	13.503	0.000000
Error	38336683	76	504430	n/a	n/a

S – sum-of-squares, DF – degrees of freedom, MS – mean squares, n/a – not applicable. Results of F-tests: F – F-value and p – p-value. Significant estimates are in red.

Supplementary Table 5. Spearman correlations (r) between the locomotor activity and experimental factors

Factors	Sex	GT	Age	3G	18°C	DD	DR	LogLA
Sex	1.000							
GT	0.012	1.000						
Age	-0.013	0.009	1.000					
3G	-0.010	0.066	-0.006	1.000				
18°C	0.010	-0.071	0.017	-0.050	1.000			
DD	0.007	-0.214	0.013	0.050	**0.693**	1.000		
DR	0.014	0.043	0.009	0.071	-0.063	**0.290**	1.000	
LogLA	-0.046	**0.233**	-0.006	-0.018	**-0.476**	**-0.610**	-0.104	1.000

Genotype (GT), combination of rapamycin, berberine and fucoxanthin (3G), low ambient temperature (18°C), maintaining in the dark (DD), dietary restriction (DR), locomotor activity (LA). Significant differences ($p<10^{-17}$) in bold.

Supplementary Table 6. Bayesian ANOVA for the locomotor activity of flies maintained in the dark and low ambient temperature taking into account genotype, sex, and age factors

Models	P(M)	P(M|data)	BF_M	BF_{10}	error %
**GT + DD + 18°C**	**0.05**	**0.54**	**22.66**	**1**	
**SEX + GT + DD + T18**	**0.05**	**0.22**	**5.34**	**0.4**	**2.76**
GT + DD	0.05	0.09	1.83	0.16	2.24
SEX + GT + DD + 18°C + SEX×GT	0.05	0.04	0.89	0.08	6.21
SEX + GT + DD	0.05	0.04	0.78	0.07	7.21
DD + 18°C	0.05	0.03	0.52	0.05	2.17
DD	0.05	0.02	0.31	0.03	16.89
SEX + DD + 18°C	0.05	$9.64×10^{-3}$	0.18	0.02	2.19
SEX + GT + DD + SEX×GT	0.05	$7.70×10^{-3}$	0.15	0.01	7.46
SEX + DD	0.05	$5.27×10^{-3}$	0.1	$9.69×10^{-3}$	3.26
GT + 18°C	0.05	$3.61×10^{-77}$	$6.87×10^{-76}$	$6.64×10^{-77}$	3.9
SEX + GT + T18	0.05	$1.03×10^{-77}$	$1.95×10^{-76}$	$1.89×10^{-77}$	2.95
SEX + GT + 18°C + SEX × GT	0.05	$1.69×10^{-78}$	$3.21×10^{-77}$	$3.10×10^{-78}$	2.83
18°C	0.05	$1.61×10^{-87}$	$3.05×10^{-86}$	$2.95×10^{-87}$	1.82
SEX + 18°C	0.05	$3.69×10^{-88}$	$7.01×10^{-87}$	$6.79×10^{-88}$	2.02
GT	0.05	$3.15×10^{-163}$	$5.99×10^{-162}$	$5.79×10^{-163}$	1.78
SEX + GT	0.05	$1.01×10^{-163}$	$1.91×10^{-162}$	$1.85×10^{-163}$	4.43
SEX + GT + SEX×GT	0.05	$1.10×10^{-164}$	$2.08×10^{-163}$	$2.01×10^{-164}$	6.25
Null model (incl. AGE)	0.05	$3.28×10^{-175}$	$6.22×10^{-174}$	$6.02×10^{-175}$	1.49
SEX	0.05	$7.95×10^{-176}$	$1.51×10^{-174}$	$1.46×10^{-175}$	2.35

Factors: low ambient temperature (18°C), maintaining in the dark (DD), genotype (GT). P(M) – prior model probability, P(M|data) – posterior model probabilities, BF_M – change from prior to posterior model odds, BF_{10} – Bayes factor. Covariates: DD and 18°C. Categorical factors: GT and SEX. Random factor: AGE. The most probable models are in bold.

Supplementary Table 7. Effect size on locomotor activity of sex, age, genotype, low temperature, and darkness

Source of variation	SS	DF	MS	F	p	Partial eta-squared	Non-centrality	Observed power (alpha=0.05)
Intercept	3540.5	1	3540.5	32263.1	0.000	0.951	32263.09	1.00
18°C	1.1	1	1.1	10.13	0.001	**0.006**	10.13	0.89
DD	42.1	1	42.1	383.69	0.000000	**0.188**	383.69	1.00
AGE	0.5	1	0.5	4.91	0.027	**0.003**	4.91	0.60
GT	1.2	1	1.2	11.18	0.001	**0.007**	11.18	0.92
SEX	0.4	1	0.4	4.05	0.044	**0.002**	4.05	0.52
GT×SEX	0.2	1	0.2	1.96	0.162	0.001	1.96	0.29
Error	181.7	1656	0.1	n/a	n/a	n/a	n/a	n/a

Factors: low ambient temperature (18°C), maintaining in the dark (DD), genotype (GT). Sum-of-squares (SS), degrees of freedom (DF), mean squares (MS), n/a – not applicable. Results of F-tests: F-value (F) and p-value (p). Covariates: 18°C, DD, and Age. Categorical factors: Genotype and Sex. Significant differences ($p<0.05$) in red. The largest effect size is in bold. Grey cells designate main effects, blue cells – minimal effects.

Supplementary Table 8. Analysis of contribution of genotype, maintaining conditions, age, and sex to stress resistance using four-way ANOVA

Source of variation	SS	DF	MS	F	p	Partial eta-squared	Non-centrality	Observed power (alpha=0.05)
Intercept	1401100	1	1401100	12383.07	0	0.853	12383.07	1
{1}GT	7110	1	7110	62.83	0	**0.028**	62.83	1
{2}COND	544	1	544	4.81	0.028	0.002	4.81	0.591705
{3}AGE	307315	2	153657	1358.04	0	**0.559**	2716.08	1
{4}SEX	16747	1	16747	148.01	0	**0.065**	148.01	1
GT×COND	1840	1	1840	16.26	0.000057	0.008	16.26	0.980826
GT×AGE	23370	2	11685	103.28	0	**0.088**	206.55	1
COND×AGE	216	2	108	0.95	0.385307	0.001	1.91	0.216553
GT×SEX	204	1	204	1.8	0.17927	0.001	1.8	0.269056
COND×SEX	4236	1	4236	37.44	0	0.017	37.44	0.999984
AGE×SEX	7938	2	3969	35.08	0	**0.032**	70.16	1
GT×COND×AGE	885	2	443	3.91	0.020	0.004	7.82	0.706621
GT×COND×SEX	2951	1	2951	26.08	0	0.012	26.08	0.999169
GT×AGE×SEX	8055	2	4027	35.59	0	**0.032**	71.19	1
COND×AGE×SEX	1780	2	890	7.87	0.0004	0.007	15.73	0.953301
1×2×3×4	277	2	138	1.22	0.29424	0.001	2.45	0.268189
Error	242360	2142	113	n/a	n/a	n/a	n/a	n/a

Genotype (GT); Maintaining conditions (COND). Control conditions: 12 h light : 12 h dark, 25°C, no substances added, normal diet. Experimental conditions: maintaining in the dark (DD), low temperature (+18°C), combination of rapamycin, berberine and fucoxanthin (3G), dietary restriction (DR). Sum-of-squares (SS), degrees of freedom (DF), mean squares (MS), n/a – not applicable. Results of F-tests: F-value (F) and p-value (p). Covariates: 18°C, DD, and Age. Categorical factors: Genotype and Sex. Significant differences (p<0.05) in red. The largest effect size is in bold. Grey cells designate main effects, blue cells – minimal effects.

Supplementary Table 9. Bayesian ANOVA for the stress resistance depending on sex, age, combination of experimental conditions, and genotype

Models	P(M)	P(M|data)	BF_M	BF_{10}	error %
**SEX + GT + COND + AGE + SEX×GT + SEX×COND + GT×COND + SEX×AGE + GT×AGE + SEX×GT×COND + SEX×GT×AGE**	**5.99e×10^{-3}**	**0.5**	**164.82**	**1**	
**SEX + GT + COND + AGE + SEX×GT + SEX×COND + GT×COND + SEX×AGE + GT×AGE + COND×AGE + SEX×GT×COND + SEX×GT×AGE + SEX×COND×AGE**	**5.99e×10^{-3}**	**0.28**	**65.41**	**0.57**	**12.42**
**SEX + GT + COND + AGE + SEX×GT + SEX×COND + GT×COND + SEX×AGE + GT×AGE + COND×AGE + SEX×GT×COND + SEX×GT×AGE + SEX×COND×AGE + GT×COND×AGE**	**5.99e×10^{-3}**	**0.16**	**32.77**	**0.33**	**11.34**
SEX + GT + COND + AGE + SEX×GT + SEX×COND + GT×COND + SEX×AGE + GT×AGE + COND×AGE + SEX×GT×COND + SEX×GT×AGE + SEX×COND×AGE + GT×COND×AGE + SEX×GT×COND×AGE	5.99e×10^{-3}	0.03	5.6	0.07	23.94
SEX + GT + COND + AGE + SEX×GT + SEX×COND + GT×COND + SEX×AGE + GT×AGE + COND×AGE + SEX×GT×COND + SEX×GT×AGE + GT×COND×AGE	5.99e×10^{-3}	0.01	1.91	0.02	45.85
SEX + GT + COND + AGE + SEX×GT + SEX×COND + GT×COND + SEX×AGE + GT×AGE + COND×AGE + SEX×GT×COND + SEX×GT×AGE	5.99e×10^{-3}	0.01	1.71	0.02	14.14
SEX + GT + COND + AGE + SEX×GT + SEX×COND + GT×COND + SEX×AGE + GT×AGE + SEX×GT×AGE	5.99e×10^{-3}	2.16e×10^{-5}	3.59e×10^{-3}	4.34e×10^{-5}	11.27
SEX + GT + COND + AGE + SEX×GT + SEX×COND + GT×COND + SEX×AGE +	5.99e×10^{-3}	1.45e×10^{-5}	2.41e×10^{-3}	2.91e×10^{-5}	22.38

GT×AGE + COND×AGE + SEX×GT×AGE + SEX×COND×AGE + GT×COND×AGE					
SEX + GT + COND + AGE + SEX×GT + SEX×COND + GT×COND + SEX×AGE + GT×AGE + COND×AGE + SEX×GT×AGE + SEX×COND×AGE	$5.99e{\times}10^{-3}$	$1.19e{\times}10^{-5}$	$1.98e{\times}10^{-3}$	$2.39e{\times}10^{-5}$	22.79
SEX + GT + COND + AGE + SEX×GT + SEX×COND + GT×COND + SEX×AGE + GT×AGE + COND×AGE + SEX×GT×AGE	$5.99e{\times}10^{-3}$	$5.12e{\times}10^{-7}$	$8.50e{\times}10^{-5}$	$1.03e{\times}10^{-6}$	12.55
SEX + GT + COND + AGE + SEX×GT + SEX×COND + GT×COND + SEX×AGE + GT×AGE + COND×AGE + SEX×GT×AGE + GT×COND×AGE	$5.99e{\times}10^{-3}$	$4.99e{\times}10^{-7}$	$8.29e{\times}10^{-5}$	$1.00e{\times}10^{-6}$	12.56
SEX + GT + COND + AGE + SEX×GT + SEX×COND + SEX×AGE + GT×AGE + SEX×GT×AGE	$5.99e{\times}10^{-3}$	$1.96e{\times}10^{-8}$	$3.26e{\times}10^{-6}$	$3.94e{\times}10^{-8}$	11.14
SEX + GT + COND + AGE + SEX×GT + SEX×COND + SEX×AGE + GT×AGE + COND×AGE + SEX×GT×AGE + SEX×COND×AGE	$5.99e{\times}10^{-3}$	$6.90e{\times}10^{-9}$	$1.14e{\times}10^{-6}$	$1.38e{\times}10^{-8}$	15.72
SEX + GT + COND + AGE + SEX×GT + SEX×COND + SEX×AGE + GT×AGE + COND×AGE + SEX×GT×AGE	$5.99e{\times}10^{-3}$	$5.29e{\times}10^{-10}$	$8.77e{\times}10^{-8}$	$1.06e{\times}10^{-9}$	12.62
SEX + GT + COND + AGE + SEX×GT + GT×COND + SEX×AGE + GT×AGE + SEX×GT×AGE	$5.99e{\times}10^{-3}$	$2.62e{\times}10^{-11}$	$4.36e{\times}10^{-9}$	$5.27e{\times}10^{-11}$	11.71
SEX + GT + COND + AGE + SEX×GT + GT×COND + SEX×AGE + GT×AGE + COND×AGE + SEX×GT×AGE + GT×COND×AGE	$5.99e{\times}10^{-3}$	$1.22e{\times}10^{-12}$	$2.03e{\times}10^{-10}$	$2.46e{\times}10^{-12}$	12.81
SEX + GT + COND + AGE + SEX×GT + GT×COND + SEX×AGE + GT×AGE + COND×AGE + SEX×GT×AGE	$5.99e{\times}10^{-3}$	$7.04e{\times}10^{-13}$	$1.17e{\times}10^{-10}$	$1.41e{\times}10^{-12}$	11.19
SEX + GT + COND + AGE + SEX×GT + SEX×COND + GT×COND + SEX×AGE + GT×AGE + SEX×GT×COND	$5.99e{\times}10^{-3}$	$3.83e{\times}10^{-14}$	$6.36e{\times}10^{-12}$	$7.69e{\times}10^{-14}$	14.39

SEX + GT + COND + AGE + SEX×GT + SEX×AGE + GT×AGE + SEX×GT×AGE	$5.99e\times10^{-3}$	$2.58e\times10^{-14}$	$4.28e\times10^{-12}$	$5.17e\times10^{-14}$	13.99
SEX + GT + AGE + SEX×GT + SEX×AGE + GT×AGE + SEX×GT×AGE	$5.99e\times10^{-3}$	$1.92e\times10^{-14}$	$3.18e\times10^{-12}$	$3.84e\times10^{-14}$	10.46
SEX + GT + COND + AGE + SEX×GT + SEX×COND + GT×COND + SEX×AGE + GT×AGE + COND×AGE + SEX×GT×COND + SEX×COND×AGE	$5.99e\times10^{-3}$	$1.50e\times10^{-14}$	$2.50e\times10^{-12}$	$3.02e\times10^{-14}$	14.13
SEX + GT + COND + AGE + SEX×GT + SEX×COND + GT×COND + SEX×AGE + GT×AGE + COND×AGE + SEX×GT×COND + SEX×COND×AGE + GT×COND×AGE	$5.99e\times10^{-3}$	$1.12e\times10^{-14}$	$1.85e\times10^{-12}$	$2.24e\times10^{-14}$	11.33
SEX + GT + COND + AGE + SEX×GT + SEX×AGE + GT×AGE + COND×AGE + SEX×GT×AGE	$5.99e\times10^{-3}$	$7.20e\times10^{-16}$	$1.20e\times10^{-13}$	$1.44e\times10^{-15}$	11.49
SEX + GT + COND + AGE + SEX×GT + SEX×COND + GT×COND + SEX×AGE + GT×AGE + COND×AGE + SEX×GT×COND	$5.99e\times10^{-3}$	$5.34e\times10^{-16}$	$8.87e\times10^{-14}$	$1.07e\times10^{-15}$	14.08
SEX + GT + COND + AGE + SEX×GT + SEX×COND + GT×COND + SEX×AGE + GT×AGE + COND×AGE + SEX×GT×COND + GT×COND×AGE	$5.99e\times10^{-3}$	$3.74e\times10^{-16}$	$6.21e\times10^{-14}$	$7.51e\times10^{-16}$	10.49
SEX + GT + COND + AGE + SEX×COND + GT×COND + SEX×AGE + GT×AGE	$5.99e\times10^{-3}$	$3.35e\times10^{-17}$	$5.56e\times10^{-15}$	$6.72e\times10^{-17}$	10.3
SEX + GT + COND + AGE + SEX×COND + GT×COND + SEX×AGE + GT×AGE + COND×AGE + SEX×COND×AGE + GT×COND×AGE	$5.99e\times10^{-3}$	$1.40e\times10^{-17}$	$2.32e\times10^{-15}$	$2.81e\times10^{-17}$	10.98
SEX + GT + COND + AGE + SEX×COND + GT×COND + SEX×AGE + GT×AGE + COND×AGE + SEX×COND×AGE	$5.99e\times10^{-3}$	$1.09e\times10^{-17}$	$1.81e\times10^{-15}$	$2.19e\times10^{-17}$	12.79
SEX + GT + COND + AGE + SEX×GT + SEX×COND + GT×COND + SEX×AGE + GT×AGE	$5.99e\times10^{-3}$	$9.63e\times10^{-18}$	$1.60e\times10^{-15}$	$1.93e\times10^{-17}$	55.36

SEX + GT + COND + AGE + SEX×GT + SEX×COND + GT×COND + SEX×AGE + GT×AGE + COND×AGE + SEX×COND×AGE + GT×COND×AGE	$5.99e{\times}10^{-3}$	$2.30e{\times}10^{-18}$	$3.81e{\times}10^{-16}$	$4.61e{\times}10^{-18}$	22.33
SEX + GT + COND + AGE + SEX×GT + SEX×COND + GT×COND + SEX×AGE + GT×AGE + COND×AGE + SEX×COND×AGE	$5.99e{\times}10^{-3}$	$1.12e{\times}10^{-18}$	$1.87e{\times}10^{-16}$	$2.26e{\times}10^{-18}$	15.41
SEX + GT + COND + AGE + SEX×COND + GT×COND + SEX×AGE + GT×AGE + COND×AGE + GT×COND×AGE	$5.99e{\times}10^{-3}$	$9.41e{\times}10^{-19}$	$1.56e{\times}10^{-16}$	$1.89e{\times}10^{-18}$	11.09
SEX + GT + COND + AGE + SEX×COND + GT×COND + SEX×AGE + GT×AGE + COND×AGE	$5.99e{\times}10^{-3}$	$6.61e{\times}10^{-19}$	$1.10e{\times}10^{-16}$	$1.33e{\times}10^{-18}$	11.64
SEX + GT + COND + AGE + SEX×GT + SEX×COND + GT×COND + SEX×AGE + GT×AGE + COND×AGE + GT×COND×AGE	$5.99e{\times}10^{-3}$	$2.78e{\times}10^{-19}$	$4.62e{\times}10^{-17}$	$5.58e{\times}10^{-19}$	58.89
SEX + GT + COND + AGE + SEX×GT + SEX×COND + GT×COND + SEX×AGE + GT×AGE + COND×AGE	$5.99e{\times}10^{-3}$	$1.12e{\times}10^{-19}$	$1.86e{\times}10^{-17}$	$2.24e{\times}10^{-19}$	27.95
SEX + GT + COND + AGE + SEX×COND + SEX×AGE + GT×AGE	$5.99e{\times}10^{-3}$	$4.28e{\times}10^{-20}$	$7.10e{\times}10^{-18}$	$8.59e{\times}10^{-20}$	10.48
SEX + GT + COND + AGE + SEX×COND + SEX×AGE + GT×AGE + COND×AGE + SEX×COND×AGE	$5.99e{\times}10^{-3}$	$7.97e{\times}10^{-21}$	$1.32e{\times}10^{-18}$	$1.60e{\times}10^{-20}$	11.82
SEX + GT + COND + AGE + SEX×GT + SEX×COND + SEX×AGE + GT×AGE	$5.99e{\times}10^{-3}$	$3.93e{\times}10^{-21}$	$6.53e{\times}10^{-19}$	$7.89e{\times}10^{-21}$	10.26
SEX + GT + COND + AGE + SEX×GT + SEX×COND + SEX×AGE + GT×AGE + COND×AGE + SEX×COND×AGE	$5.99e{\times}10^{-3}$	$1.10e{\times}10^{-21}$	$1.82e{\times}10^{-19}$	$2.20e{\times}10^{-21}$	37.52
SEX + GT + COND + AGE + SEX×COND + SEX×AGE + GT×AGE + COND×AGE	$5.99e{\times}10^{-3}$	$8.74e{\times}10^{-22}$	$1.45e{\times}10^{-19}$	$1.75e{\times}10^{-21}$	10.98
SEX + GT + COND + AGE + GT×COND + SEX×AGE + GT×AGE	$5.99e{\times}10^{-3}$	$1.04e{\times}10^{-22}$	$1.72e{\times}10^{-20}$	$2.08e{\times}10^{-22}$	10.16

SEX + GT + COND + AGE + SEX×GT + SEX×COND + SEX×AGE + GT×AGE + COND×AGE	$5.99e\times10^{-3}$	$8.85e\times10^{-23}$	$1.47e\times10^{-20}$	$1.78e\times10^{-22}$	11.07
SEX + GT + COND + AGE + SEX×GT + GT×COND + SEX×AGE + GT×AGE	$5.99e\times10^{-3}$	$1.24e\times10^{-23}$	$2.06e\times10^{-21}$	$2.49e\times10^{-23}$	10.47
SEX + GT + COND + AGE + GT×COND + SEX×AGE + GT×AGE + COND×AGE + GT×COND×AGE	$5.99e\times10^{-3}$	$4.46e\times10^{-24}$	$7.40e\times10^{-22}$	8.95e×10-24	10.24
SEX + GT + COND + AGE + GT×COND + SEX×AGE + GT×AGE + COND×AGE	$5.99e\times10^{-3}$	$2.34e\times10^{-24}$	$3.88e\times10^{-22}$	4.70e×10-24	10.71
SEX + GT + COND + AGE + SEX×GT + GT×COND + SEX×AGE + GT×AGE + COND×AGE + GT×COND×AGE	$5.99e\times10^{-3}$	$6.98e\times10^{-25}$	$1.16e\times10^{-22}$	1.40e×10-24	15.76
SEX + GT + COND + AGE + SEX×GT + GT×COND + SEX×AGE + GT×AGE + COND×AGE	$5.99e\times10^{-3}$	$2.74e\times10^{-25}$	$4.55e\times10^{-23}$	$5.50e\times10^{-25}$	11.35
SEX + GT + AGE + SEX×AGE + GT×AGE	$5.99e\times10^{-3}$	$1.38e\times10^{-25}$	$2.29e\times10^{-23}$	$2.77e\times10^{-25}$	10.49
SEX + GT + COND + AGE + SEX×AGE + GT×AGE	$5.99e\times10^{-3}$	$1.15e\times10^{-25}$	$1.91e\times10^{-23}$	$2.31e\times10^{-25}$	10.13
SEX + GT + COND + AGE + SEX×GT + SEX×COND + GT×COND + GT×AGE + SEX×GT×COND	$5.99e\times10^{-3}$	$1.05e\times10^{-25}$	$1.74e\times10^{-23}$	$2.10e\times10^{-25}$	17.09
SEX + GT + AGE + SEX×GT + SEX×AGE + GT×AGE	$5.99e\times10^{-3}$	$1.65e\times10^{-26}$	2.74e×10-24	$3.32e\times10^{-26}$	10.42
SEX + GT + COND + AGE + SEX×GT + SEX×AGE + GT×AGE	$5.99e\times10^{-3}$	$1.37e\times10^{-26}$	2.28e×10-24	$2.76e\times10^{-26}$	10.52
SEX + GT + COND + AGE + SEX×AGE + GT×AGE + COND×AGE	$5.99e\times10^{-3}$	$3.04e\times10^{-27}$	$5.05e\times10^{-25}$	$6.11e\times10^{-27}$	10.38
SEX + GT + COND + AGE + SEX×GT + SEX×COND + GT×COND + GT×AGE + COND×AGE + SEX×GT×COND	$5.99e\times10^{-3}$	$1.71e\times10^{-27}$	$2.84e\times10^{-25}$	$3.43e\times10^{-27}$	13.73
SEX + GT + COND + AGE + SEX×GT + SEX×COND + GT×COND + GT×AGE + COND×AGE +	$5.99e\times10^{-3}$	$5.66e\times10^{-28}$	$9.40e\times10^{-26}$	$1.14e\times10^{-27}$	18.48

SEX×GT×COND + GT×COND×AGE					
SEX + GT + COND + AGE + SEX×GT + SEX×AGE + GT×AGE + COND×AGE	5.99e×10^{-3}	3.33e×10^{-28}	5.52e×10^{-26}	6.68e×10^{-28}	12.06
SEX + GT + COND + AGE + SEX×COND + GT×COND + GT×AGE	5.99e×10^{-3}	2.14e×10^{-28}	3.55e×10^{-26}	4.29e×10^{-28}	10.36
SEX + GT + COND + AGE + SEX×GT + SEX×COND + GT×COND + GT×AGE	5.99e×10^{-3}	3.58e×10^{-29}	5.94e×10^{-27}	7.18e×10^{-29}	35.15
SEX + GT + COND + AGE + SEX×COND + GT×COND + GT×AGE + COND×AGE	5.99e×10^{-3}	5.31e×10^{-30}	8.81e×10^{-28}	1.07e×10^{-29}	11.78
SEX + GT + COND + AGE + SEX×COND + GT×COND + GT×AGE + COND×AGE + GT×COND×AGE	5.99e×10^{-3}	2.32e×10^{-30}	3.86e×10^{-28}	4.66e×10^{-30}	14.2
SEX + GT + COND + AGE + SEX×COND + GT×AGE	5.99e×10^{-3}	1.02e×10^{-30}	1.70e×10^{-28}	2.06e×10^{-30}	10.27
SEX + GT + COND + AGE + SEX×GT + SEX×COND + GT×COND + GT×AGE + COND×AGE	5.99e×10^{-3}	4.21e×10^{-31}	6.99e×10^{-29}	8.46e×10^{-31}	11.07
SEX + GT + COND + AGE + SEX×GT + SEX×COND + GT×COND + GT×AGE + COND×AGE + GT×COND×AGE	5.99e×10^{-3}	2.40e×10^{-31}	3.99e×10^{-29}	4.82e×10^{-31}	13.1
SEX + GT + COND + AGE + SEX×GT + SEX×COND + GT×AGE	5.99e×10^{-3}	9.14e×10^{-32}	1.52e×10^{-29}	1.83e×10^{-31}	10.64
SEX + GT + COND + AGE + SEX×COND + GT×AGE + COND×AGE	5.99e×10^{-3}	2.64e×10^{-32}	4.38e×10^{-30}	5.30e×10^{-32}	10.7
SEX + GT + COND + AGE + GT×COND + GT×AGE	5.99e×10^{-3}	5.19e×10^{-33}	8.61e×10^{-31}	1.04e×10^{-32}	10.23
SEX + GT + COND + AGE + SEX×GT + SEX×COND + GT×AGE + COND×AGE	5.99e×10^{-3}	2.20e×10^{-33}	3.65e×10^{-31}	4.41e×10^{-33}	10.3
SEX + GT + COND + AGE + SEX×GT + GT×COND + GT×AGE	5.99e×10^{-3}	4.54e×10^{-34}	7.54e×10^{-32}	9.12e×10^{-34}	10.26
SEX + GT + COND + AGE + GT×COND + GT×AGE + COND×AGE	5.99e×10^{-3}	1.35e×10^{-34}	2.23e×10^{-32}	2.70e×10^{-34}	13.03

SEX + GT + COND + AGE + GT×COND + GT×AGE + COND×AGE + GT×COND×AGE	5.99e×10^{-3}	8.36e×10^{-35}	1.39e×10^{-32}	1.68e×10^{-34}	10.37
SEX + GT + COND + AGE + GT×AGE	5.99e×10^{-3}	1.97e×10^{-35}	3.27e×10^{-33}	3.95e×10^{-35}	11.15
SEX + GT + AGE + GT×AGE	5.99e×10^{-3}	1.67e×10^{-35}	2.77e×10^{-33}	3.35e×10^{-35}	9.94
SEX + GT + COND + AGE + SEX×GT + GT×COND + GT×AGE + COND×AGE	5.99e×10^{-3}	1.21e×10^{-35}	2.01e×10^{-33}	2.44e×10^{-35}	10.43
SEX + GT + COND + AGE + SEX×GT + GT×COND + GT×AGE + COND×AGE + GT×COND×AGE	5.99e×10^{-3}	1.06e×10^{-35}	1.76e×10^{-33}	2.13e×10^{-35}	13.46
SEX + GT + COND + AGE + SEX×GT + GT×AGE	5.99e×10^{-3}	2.04e×10^{-36}	3.38e×10^{-34}	4.09e×10^{-36}	12.1
SEX + GT + AGE + SEX×GT + GT×AGE	5.99e×10^{-3}	1.74e×10^{-36}	2.89e×10^{-34}	3.49e×10^{-36}	10.26
SEX + GT + COND + AGE + GT×AGE + COND×AGE	5.99e×10^{-3}	6.35e×10^{-37}	1.05e×10^{-34}	1.28e×10^{-36}	10.61
SEX + GT + COND + AGE + SEX×GT + GT×AGE + COND×AGE	5.99e×10^{-3}	7.62e×10^{-38}	1.26e×10^{-35}	1.53e×10^{-37}	27.67
SEX + GT + COND + AGE + SEX×GT + SEX×COND + GT×COND + SEX×AGE + COND×AGE + SEX×GT×COND + SEX×COND×AGE	5.99e×10^{-3}	3.37e×10^{-55}	5.59e×10^{-5}3	6.76e×10^{-55}	18.82
SEX + GT + COND + AGE + SEX×GT + SEX×COND + GT×COND + SEX×AGE + SEX×GT×COND	5.99e×10^{-3}	1.60e×10^{-55}	2.66e×10^{-5}3	3.22e×10^{-55}	12.51
SEX + GT + COND + AGE + SEX×GT + SEX×COND + GT×COND + SEX×AGE + COND×AGE + SEX×GT×COND	5.99e×10^{-3}	2.04e×10^{-57}	3.38e×10^{-55}	4.09e×10^{-57}	11.56
GT + COND + AGE + GT×COND + GT×AGE	5.99e×10^{-3}	1.03e×10^{-57}	1.71e×10^{-55}	2.07e×10^{-57}	10.32
SEX + GT + COND + AGE + SEX×COND + GT×COND + SEX×AGE + COND×AGE + SEX×COND×AGE	5.99e×10^{-3}	1.94e×10^{-58}	3.23e×10^{-56}	3.90e×10^{-58}	12.29
SEX + GT + COND + AGE + SEX×COND + GT×COND + SEX×AGE	5.99e×10^{-3}	1.87e×10^{-58}	3.11e×10^{-56}	3.76e×10^{-58}	10.73

GT + COND + AGE + GT×COND + GT×AGE + COND×AGE + GT×COND×AGE	$5.99e\times10^{-3}$	$5.59e\times10^{-59}$	$9.27e\times10^{-57}$	$1.12e\times10^{-58}$	12.04
SEX + GT + COND + AGE + SEX×GT + SEX×COND + GT×COND + SEX×AGE + COND×AGE + SEX×COND×AGE	$5.99e\times10^{-3}$	$1.95e\times10^{-59}$	$3.23e\times10^{-57}$	$3.90e\times10^{-59}$	18.64
GT + COND + AGE + GT×COND + GT×AGE + COND×AGE	$5.99e\times10^{-3}$	$1.94e\times10^{-59}$	$3.22e\times10^{-57}$	$3.89e\times10^{-59}$	10.2
SEX + GT + COND + AGE + SEX×GT + SEX×COND + GT×COND + SEX×AGE	$5.99e\times10^{-3}$	$1.72e\times10^{-59}$	$2.85e\times10^{-57}$	$3.45e\times10^{-59}$	17.99
SEX + GT + COND + AGE + SEX×COND + GT×COND + SEX×AGE + COND×AGE	$5.99e\times10^{-3}$	$3.70e\times10^{-60}$	$6.13e\times10^{-58}$	$7.42e\times10^{-60}$	28.07
SEX + GT + COND + AGE + SEX×COND + SEX×AGE	$5.99e\times10^{-3}$	$2.70e\times10^{-60}$	$4.48e\times10^{-58}$	$5.42e\times10^{-60}$	11.57
GT + AGE + GT×AGE	$5.99e\times10^{-3}$	$2.45e\times10^{-60}$	$4.07e\times10^{-58}$	$4.93e\times10^{-60}$	10.41
SEX + GT + COND + AGE + SEX×COND + SEX×AGE + COND×AGE + SEX×COND×AGE	$5.99e\times10^{-3}$	$1.70e\times10^{-60}$	$2.82e\times10^{-58}$	$3.41e\times10^{-60}$	10.36
GT + COND + AGE + GT×AGE	$5.99e\times10^{-3}$	$1.66e\times10^{-60}$	$2.75e\times10^{-58}$	$3.33e\times10^{-60}$	10.2
SEX + GT + COND + AGE + SEX×GT + SEX×COND + GT×COND + SEX×AGE + COND×AGE	$5.99e\times10^{-3}$	$2.47e\times10^{-61}$	$4.10e\times10^{-59}$	$4.96e\times10^{-61}$	13.38
SEX + GT + COND + AGE + SEX×GT + SEX×COND + SEX×AGE	$5.99e\times10^{-3}$	$2.38e\times10^{-61}$	$3.96e\times10^{-59}$	$4.78e\times10^{-61}$	14.81
SEX + GT + COND + AGE + SEX×GT + SEX×COND + SEX×AGE + COND×AGE + SEX×COND×AGE	$5.99e\times10^{-3}$	$1.54e\times10^{-61}$	$2.56e\times10^{-59}$	$3.09e\times10^{-61}$	14.46
SEX + GT + COND + AGE + SEX×COND + SEX×AGE + COND×AGE	$5.99e\times10^{-3}$	$4.55e\times10^{-62}$	$7.55e\times10^{-60}$	$9.13e\times10^{-62}$	11.22
GT + COND + AGE + GT×AGE + COND×AGE	$5.99e\times10^{-3}$	$3.78e\times10^{-62}$	$6.27e\times10^{-60}$	$7.58e\times10^{-62}$	14.48
SEX + GT + COND + AGE + GT×COND + SEX×AGE	$5.99e\times10^{-3}$	$2.28e\times10^{-62}$	$3.79e\times10^{-60}$	$4.58e\times10^{-62}$	12.45

SEX + GT + COND + AGE + SEX×GT + SEX×COND + SEX×AGE + COND×AGE	$5.99e{\times}10^{-3}$	$4.61e{\times}10^{-63}$	$7.66e{\times}10^{-61}$	$9.26e{\times}10^{-63}$	16.35
SEX + GT + COND + AGE + SEX×GT + GT×COND + SEX×AGE	$5.99e{\times}10^{-3}$	$1.88e{\times}10^{-63}$	$3.11e{\times}10^{-61}$	$3.76e{\times}10^{-63}$	10.63
SEX + GT + AGE + SEX×AGE	$5.99e{\times}10^{-3}$	$6.91e{\times}10^{-64}$	$1.15e{\times}10^{-61}$	$1.39e{\times}10^{-63}$	11.05
SEX + GT + COND + AGE + GT×COND + SEX×AGE + COND×AGE	$5.99e{\times}10^{-3}$	$4.08e{\times}10^{-64}$	$6.78e{\times}10^{-62}$	$8.20e{\times}10^{-64}$	16
SEX + GT + AGE + SEX×GT + SEX×AGE	$5.99e{\times}10^{-3}$	$3.29e{\times}10^{-64}$	$5.46e{\times}10^{-62}$	$6.60e{\times}10^{-64}$	83.68
SEX + GT + COND + AGE + SEX×AGE	$5.99e{\times}10^{-3}$	$2.62e{\times}10^{-64}$	$4.35e{\times}10^{-62}$	$5.26e{\times}10^{-64}$	10.14
SEX + GT + COND + AGE + SEX×GT + GT×COND + SEX×AGE + COND×AGE	$5.99e{\times}10^{-3}$	$3.60e{\times}10^{-65}$	$5.98e{\times}10^{-63}$	$7.23e{\times}10^{-65}$	13.67
SEX + GT + COND + AGE + SEX×GT + SEX×AGE	$5.99e{\times}10^{-3}$	$2.30e{\times}10^{-65}$	$3.82e{\times}10^{-63}$	$4.62e{\times}10^{-65}$	10.83
SEX + GT + COND + AGE + SEX×GT + SEX×COND + GT×COND + SEX×GT×COND	$5.99e{\times}10^{-3}$	$1.64e{\times}10^{-65}$	$2.72e{\times}10^{-63}$	$3.29e{\times}10^{-65}$	10.34
SEX + GT + COND + AGE + SEX×AGE + COND×AGE	$5.99e{\times}10^{-3}$	$4.84e{\times}10^{-66}$	$8.03e{\times}10^{-64}$	$9.71e{\times}10^{-66}$	10.13
SEX + GT + COND + AGE + SEX×GT + SEX×AGE + COND×AGE	$5.99e{\times}10^{-3}$	$4.33e{\times}10^{-67}$	$7.18e{\times}10^{-65}$	$8.69e{\times}10^{-67}$	10.78
SEX + GT + COND + AGE + SEX×GT + SEX×COND + GT×COND + COND×AGE + SEX×GT×COND	$5.99e{\times}10^{-3}$	$3.03e{\times}10^{-67}$	$5.02e{\times}10^{-65}$	$6.07e{\times}10^{-67}$	18.66
SEX + GT + COND + AGE + SEX×COND + GT×COND	$5.99e{\times}10^{-3}$	$5.95e{\times}10^{-68}$	$9.88e{\times}10^{-66}$	$1.19e{\times}10^{-67}$	29.69
SEX + COND + AGE + SEX×COND + SEX×AGE	$5.99e{\times}10^{-3}$	$2.77e{\times}10^{-68}$	$4.60e{\times}10^{-66}$	$5.56e{\times}10^{-68}$	11.13
SEX + GT + COND + AGE + SEX×GT + SEX×COND + GT×COND	$5.99e{\times}10^{-3}$	$8.45e{\times}10^{-69}$	$1.40e{\times}10^{-66}$	$1.70e{\times}10^{-68}$	62.25
SEX + COND + AGE + SEX×COND + SEX×AGE + COND×AGE + SEX×COND×AGE	$5.99e{\times}10^{-3}$	$6.51e{\times}10^{-69}$	$1.08e{\times}10^{-66}$	$1.31e{\times}10^{-68}$	19.83
SEX + GT + COND + AGE + SEX×COND	$5.99e{\times}10^{-3}$	$1.86e{\times}10^{-69}$	$3.10e{\times}10^{-67}$	$3.74e{\times}10^{-69}$	10.06

SEX + GT + COND + AGE + SEX×COND + GT×COND + COND×AGE	$5.99e\times10^{-3}$	$7.37e\times10^{-70}$	$1.22e\times10^{-67}$	$1.48e\times10^{-69}$	10.78
SEX + COND + AGE + SEX×COND + SEX×AGE + COND×AGE	$5.99e\times10^{-3}$	$5.01e\times10^{-70}$	$8.32e\times10^{-68}$	$1.01e\times10^{-69}$	10.31
SEX + GT + COND + AGE + SEX×GT + SEX×COND	$5.99e\times10^{-3}$	$1.29e\times10^{-70}$	$2.14e\times10^{-68}$	$2.59e\times10^{-70}$	9.96
SEX + GT + COND + AGE + SEX×GT + SEX×COND + GT×COND + COND×AGE	$5.99e\times10^{-3}$	$5.69e\times10^{-71}$	$9.44e\times10^{-69}$	$1.14e\times10^{-70}$	10.67
SEX + GT + COND + AGE + SEX×COND + COND×AGE	$5.99e\times10^{-3}$	$3.95e\times10^{-71}$	$6.56e\times10^{-69}$	$7.93e\times10^{-71}$	10.59
SEX + GT + COND + AGE + GT×COND	$5.99e\times10^{-3}$	$2.54e\times10^{-71}$	$4.21e\times10^{-69}$	$5.09e\times10^{-71}$	10.26
SEX + GT + COND + AGE + SEX×GT + SEX×COND + COND×AGE	$5.99e\times10^{-3}$	$2.72e\times10^{-72}$	$4.51e\times10^{-70}$	$5.45e\times10^{-72}$	10.17
SEX + AGE + SEX×AGE	$5.99e\times10^{-3}$	$2.41e\times10^{-72}$	$4.00e\times10^{-70}$	$4.84e\times10^{-72}$	10.26
SEX + GT + COND + AGE + SEX×GT + GT×COND	$5.99e\times10^{-3}$	$2.03e\times10^{-72}$	$3.37e\times10^{-70}$	$4.07e\times10^{-72}$	10.16
SEX + GT + AGE	$5.99e\times10^{-3}$	$1.79e\times10^{-72}$	$2.97e\times10^{-70}$	$3.59e\times10^{-72}$	9.95
SEX + COND + AGE + SEX×AGE	$5.99e\times10^{-3}$	$1.36e\times10^{-72}$	$2.26e\times10^{-70}$	$2.74e\times10^{-72}$	11.97
SEX + GT + COND + AGE	$5.99e\times10^{-3}$	$1.04e\times10^{-72}$	$1.73e\times10^{-70}$	$2.10e\times10^{-72}$	10.84
SEX + GT + COND + AGE + GT×COND + COND×AGE	$5.99e\times10^{-3}$	$5.77e\times10^{-73}$	$9.58e\times10^{-71}$	$1.16e\times10^{-72}$	11.23
SEX + GT + AGE + SEX×GT	$5.99e\times10^{-3}$	$1.43e\times10^{-73}$	$2.38e\times10^{-71}$	$2.88e\times10^{-73}$	9.96
SEX + GT + COND + AGE + SEX×GT	$5.99e\times10^{-3}$	$7.86e\times10^{-74}$	$1.30e\times10^{-71}$	$1.58e\times10^{-73}$	10.31
SEX + GT + COND + AGE + SEX×GT + GT×COND + COND×AGE	$5.99e\times10^{-3}$	$4.19e\times10^{-74}$	$6.96e\times10^{-72}$	$8.42e\times10^{-74}$	10.34
SEX + COND + AGE + SEX×AGE + COND×AGE	$5.99e\times10^{-3}$	$3.12e\times10^{-74}$	$5.18e\times10^{-72}$	$6.26e\times10^{-74}$	16.4
SEX + GT + COND + AGE + COND×AGE	$5.99e\times10^{-3}$	$2.45e\times10^{-74}$	$4.07e\times10^{-72}$	$4.92e\times10^{-74}$	12.13
SEX + GT + COND + AGE + SEX×GT + COND×AGE	$5.99e\times10^{-3}$	$1.78e\times10^{-75}$	$2.96e\times10^{-73}$	$3.57e\times10^{-75}$	10.33
SEX + COND + AGE + SEX×COND	$5.99e\times10^{-3}$	$2.19e\times10^{-76}$	$3.63e\times10^{-74}$	$4.40e\times10^{-76}$	10.98

SEX + COND + AGE + SEX×COND + COND×AGE	5.99e×10^{-3}	5.12e×10^{-78}	8.50e×10^{-76}	1.03e×10^{-77}	10.93
SEX + AGE	5.99e×10^{-3}	6.71e×10^{-80}	1.11e×10^{-77}	1.35e×10^{-79}	9.78
SEX + COND + AGE	5.99e×10^{-3}	4.83e×10^{-80}	8.01e×10^{-78}	9.69e×10^{-80}	10.07
SEX + COND + AGE + COND×AGE	5.99e×10^{-3}	1.33e×10^{-81}	2.20e×10^{-79}	2.66e×10^{-81}	10.26
GT + COND + AGE + GT×COND	5.99e×10^{-3}	1.64e×10^{-91}	2.72e×10^{-8}9	3.29e×10^{-91}	10.18
GT + AGE	5.99e×10^{-3}	6.74e×10^{-93}	1.12e×10^{-9}0	1.35e×10^{-92}	10.08
GT + COND + AGE + GT×COND + COND×AGE	5.99e×10^{-3}	2.84e×10^{-93}	4.71e×10^{-91}	5.70e×10^{-93}	10.73
GT + COND + AGE	5.99e×10^{-3}	2.51e×10^{-93}	4.16e×10^{-91}	5.03e×10^{-93}	9.9
GT + COND + AGE + COND×AGE	5.99e×10^{-3}	4.72e×10^{-95}	7.84e×10^{-93}	9.48e×10^{-9}5	10.31
AGE	5.99e×10^{-3}	2.42e×10^{-100}	4.02e×10^{-98}	4.86e×10^{-10}0	9.7
COND + AGE	5.99e×10^{-3}	1.15e×10^{-100}	1.91e×10^{-98}	2.30e×10^{-10}0	9.94
COND + AGE + COND×AGE	5.99e×10^{-3}	2.51e×10^{-102}	4.16e×10^{-100}	5.03e×10^{-10}2	13.87
SEX + GT + COND + SEX×COND	5.99e×10^{-3}	0	0	0	14.26
SEX + GT + COND + SEX×COND + GT×COND	5.99e×10^{-3}	0	0	0	10.21
SEX + GT	5.99e×10^{-3}	0	0	0	9.76
SEX + GT + COND + SEX×GT + SEX×COND	5.99e×10^{-3}	0	0	0	12.77
SEX + COND + SEX×COND	5.99e×10^{-3}	0	0	0	9.82
SEX + GT + COND + SEX×GT + SEX×COND + GT×COND + SEX×GT×COND	5.99e×10^{-3}	0	0	0	10.71
SEX + GT + COND + SEX×GT + SEX×COND + GT×COND	5.99e×10^{-3}	0	0	0	10.48
SEX + GT + SEX×GT	5.99e×10^{-3}	0	0	0	12.42
SEX + GT + COND	5.99e×10^{-3}	0	0	0	9.86
SEX	5.99e×10^{-3}	0	0	0	9.7
SEX + GT + COND + GT×COND	5.99e×10^{-3}	0	0	0	10.15
SEX + GT + COND + SEX×GT	5.99e×10^{-3}	0	0	0	9.88
SEX + GT + COND + SEX×GT + GT×COND	5.99e×10^{-3}	0	0	0	10.05

SEX + COND	$5.99e\times10^{-3}$	0	0	0	10.06
GT	$5.99e\times10^{-3}$	0	0	0	9.7
GT + COND	$5.99e\times10^{-3}$	0	0	0	9.88
GT + COND + GT×COND	$5.99e\times10^{-3}$	0	0	0	13.22
Null model	$5.99e\times10^{-3}$	0	0	0	9.7
COND	$5.99e\times10^{-3}$	0	0	0	9.7

GT – *E(z)/w* genotype, COND –combination of experimental conditions (maintaining in the dark, low ambient temperature, a combination of rapamycin, berberine, and fucoxanthin, and dietary restriction), P(M) – prior model probability, P(M|data) – posterior model probabilities, BF_M – change from prior odds to posterior odds for each model, BF_{10} – Bayes factor.

Supplementary Table 10. The effects of sex, genotype, experimental conditions, and age on resistance to oxidative stress in flies

SEX	GT	COND	AGE	Mean (h)	±SE	n	p^{BF} (SEX)	p^{BF} (GT)	p^{BF} (COND)	p^{BF} (AGE)
♂	*w/w*	control	10	35.34	1.37	80	n/a	n/a	n/a	n/a
♂	*E(z)/w*	control	10	44.08	1.88	80	n/a	**0.000**	n/a	n/a
♂	*w/w*	control	30	15.55	0.66	96	n/a	n/a	n/a	**0.000**
♂	*E(z)/w*	control	30	21.01	0.89	80	n/a	0.196	n/a	**0.000**
♂	*w/w*	control	45	15.58	0.77	73	n/a	n/a	n/a	**0.000**
♂	*E(z)/w*	control	45	12.05	0.78	81	n/a	1.000	n/a	**0.000**
♂	*w/w*	DR, 3G, 18°C, DD	10	32.75	1.49	79	n/a	n/a	1.000	n/a
♂	*E(z)/w*	DR, 3G, 18°C, DD	10	37.35	1.68	78	n/a	1.000	**0.020**	n/a
♂	*w/w*	DR, 3G, 18°C, DD	30	15.12	0.67	76	n/a	n/a	1.000	**0.000**
♂	*E(z)/w*	DR, 3G, 18°C, DD	30	17.38	0.58	78	n/a	1.000	1.000	**0.000**
♂	*w/w*	DR, 3G, 18°C, DD	45	14.62	0.41	120	n/a	n/a	1.000	**0.000**
♂	*E(z)/w*	DR, 3G, 18°C, DD	45	15.44	0.39	96	n/a	1.000	1.000	**0.000**
♀	*w/w*	control	10	37.82	1.57	96	1.000	n/a	n/a	n/a
♀	*E(z)/w*	control	10	53.68	2.33	80	**0.000**	**0.000**	n/a	n/a
♀	*w/w*	control	30	22.82	1.09	79	**0.002**	n/a	n/a	**0.000**
♀	*E(z)/w*	control	30	16.09	0.58	79	0.981	**0.020**	n/a	**0.000**
♀	*w/w*	control	45	19.49	0.66	122	1.000	n/a	n/a	**0.000**
♀	*E(z)/w*	control	45	10.56	0.38	108	1.000	**0.000**	n/a	**0.000**
♀	*w/w*	DR, 3G, 18°C, DD	10	39.65	1.29	96	**0.006**	n/a	1.000	n/a
♀	*E(z)/w*	DR, 3G, 18°C, DD	10	61.94	2.39	79	**0.000**	**0.000**	**0.000**	n/a
♀	*w/w*	DR, 3G, 18°C, DD	30	22.59	1.05	79	**0.004**	n/a	1.000	**0.000**
♀	*E(z)/w*	DR, 3G, 18°C, DD	30	24.94	1.17	80	**0.002**	1.000	**0.000**	**0.000**
♀	*w/w*	DR, 3G, 18°C, DD	45	16.74	0.73	101	1.000	n/a	1.000	**0.000**
♀	*E(z)/w*	DR, 3G, 18°C, DD	45	17.77	0.59	150	1.000	1.000	**0.000**	**0.000**

Sex: males (♂) and females (♀); Genotype (GT); Maintaining conditions (COND). Control conditions: 12 h light : 12 h dark, 25°C, no substances added, normal diet. Experimental conditions: maintaining in the dark (DD), low temperature (+18°C), combination of rapamycin, berberine and fucoxanthin (3G), dietary restriction (DR). Mean (h) – mean survival time; ±SE – standard error. p^{BF} (SEX), p^{BF} (GT), p^{BF} (COND), p^{BF} (AGE) – p-value for Bonferroni post-hoc tests with four-way ANOVA to determine the differences of mean survival time which related to sex, genotype, maintaining conditions, and age, respectively.

Supplementary Table 11. Bayesian ANOVA for the lipid levels in the male flies

Models	P(M)	P(M|data)	BF_M	BF_{10}	error %
Null model	0.053	0.0004	0.007	1	
[GT] + [AGE]	0.053	0.407	12.33	1045.7	4.0
[GT]	0.053	0.148	3.12	379.6	0.00000006
[GT] + [AGE] + [COND] + [GT × COND]	0.053	0.105	2.12	271.0	2.4
[GT] + [AGE] + [COND]	0.053	0.099	1.97	253.8	7.3
[GT] + [AGE] + [GT × AGE]	0.053	0.066	1.28	170.5	3.4
[GT] + [COND]	0.053	0.037	0.70	95.6	0.9
[GT] + [COND] + [GT × COND]	0.053	0.034	0.63	86.4	1.6
[GT] + [AGE] + [COND] + [GT × COND] + [AGE × COND]	0.053	0.033	0.61	83.7	4.5
[GT] + [AGE] + [COND] + [AGE × COND]	0.053	0.027	0.49	68.8	2.4
[GT] + [AGE] + [COND] + [GT × AGE] + [GT × COND]	0.053	0.017	0.31	43.5	3.8
[GT] + [AGE] + [COND] + [GT × AGE]	0.053	0.014	0.27	37.3	1.7
[GT] + [AGE] + [COND] + [GT × AGE] + [GT × COND] + [AGE × COND]	0.053	0.005	0.088	12.6	2.6
[GT] + [AGE] + [COND] + [GT × AGE] + [AGE × COND]	0.053	0.004	0.076	10.9	1.9
[GT] + [AGE] + [COND] + [GT · AGE] + [GT × COND] + [AGE × COND] + [GT × AGE × COND]	0.053	0.003	0.059	8.35	10.8
[AGE]	0.053	0.001	0.019	2.74	0.03
[AGE] + [COND]	0.053	0.0003	0.005	0.67	1.4
[COND]	0.053	0.0002	0.002	0.27	0.02
[AGE] + [COND] + [AGE × COND]	0.053	0.00008	0.001	0.19	2.2

GT – genotype, COND – combination of experimental conditions (maintaining in the dark, low ambient temperature, a combination of rapamycin, berberine, and fucoxanthin, and dietary restriction), P(M) – prior model probability, P(M|data) – posterior model probabilities, BF_M – change from prior odds to posterior odds for each model, BF_{10} – Bayes factor.

Supplementary Table 12. Bayesian ANOVA for the lipid levels in the female flies

Models	P(M)	P(M|data)	BF_M	BF_{10}	error %
Null model	0.053	0.027	0.50	1	
[GT] + [AGE]	0.053	0.28	7.01	10.34	1.2
[AGE]	0.053	0.16	3.44	5.91	0.02
[GT] + [AGE] + [GT × AGE]	0.053	0.09	1.78	3.32	1.5
[GT] + [AGE] + [COND]	0.053	0.079	1.54	2.91	1.3
[GT] + [AGE] + [COND] + [AGE × COND]	0.053	0.069	1.33	2.54	1.8
[AGE] + [COND]	0.053	0.044	0.83	1.63	1.0
[GT] + [AGE] + [COND] + [GT × COND]	0.053	0.044	0.82	1.62	1.7
[GT] + [AGE] + [COND] + [GT × COND] + [AGE × COND]	0.053	0.032	0.60	1.20	3.1
[AGE] + [COND] + [AGE · COND]	0.053	0.031	0.58	1.16	1.6
[GT]	0.053	0.029	0.54	1.08	0.01
[GT] + [AGE] + [COND] + [GT × AGE]	0.053	0.025	0.46	0.92	1.6
[GT] + [AGE] + [COND] + [GT × AGE] + [AGE × COND]	0.053	0.021	0.39	0.79	2.3
[GT] + [COND]	0.053	0.019	0.34	0.68	0.9
[COND]	0.053	0.016	0.29	0.59	0.00003
[GT] + [AGE] + [COND] + [GT × AGE] + [GT × COND]	0.053	0.011	0.20	0.41	2.4
[GT] + [COND] + [GT × COND]	0.053	0.01	0.19	0.39	3.9
[GT] + [AGE] + [COND] + [GT × AGE] + [GT × COND] + [AGE × COND]	0.053	0.009	0.16	0.33	2.7
[GT] + [AGE] + [COND] + [GT × AGE] + [GT × COND] + [AGE × COND] + [GT × AGE × COND]	0.053	0.002	0.04	0.08	3.8

GT - genotype, COND – combination of experimental conditions (maintaining in the dark, low ambient temperature, a combination of rapamycin, berberine, and fucoxanthin, and dietary restriction), P(M) – prior model probability, P(M|data) – posterior model probabilities, BF_M – change from prior odds to posterior odds for each model, BF_{10} – Bayes factor.

Supplementary Table 13. Bayesian ANOVA for the lipid levels in the flies subjected to combination of experimental factors

Models	P(M)	P(M|data)	BF_M	BF_{10}	error %
Null model	0.053	0.0005	0.008	1	
[SEX] + [GT] + [AGE]	0.053	0.437	13.97	1009.7	15.8
[SEX] + [GT] + [AGE] + [SEX × GT]	0.053	0.127	2.62	293.9	2.9
[SEX] + [GT] + [AGE] + [SEX × AGE]	0.053	0.126	2.59	290.3	1.6
[SEX] + [AGE]	0.053	0.101	2.02	232.7	1.1
[SEX] + [GT] + [AGE] + [SEX × GT] + [SEX × AGE]	0.053	0.047	0.89	109.0	7.4
[SEX] + [GT] + [AGE] + [GT × AGE]	0.053	0.039	0.74	90.9	12.2
[SEX] + [AGE] + [SEX × AGE]	0.053	0.038	0.72	88.8	1.7
[GT] + [AGE]	0.053	0.031	0.57	71.3	1.7
[SEX] + [GT] + [AGE] + [SEX × AGE] + [GT × AGE]	0.053	0.013	0.23	29.2	2.1
[SEX] + [GT] + [AGE] + [SEX × GT] + [GT × AGE]	0.053	0.012	0.21	27.1	3.2
[AGE]	0.053	0.011	0.21	26.4	0.006
[SEX] + [GT]	0.053	0.004	0.080	10.2	1.3
[SEX] + [GT] + [AGE] + [SEX × GT] + [SEX × AGE] + [GT × AGE]	0.053	0.004	0.079	10.1	6.9
[GT] + [AGE] + [GT × AGE]	0.053	0.003	0.055	7.0	1.7
[SEX]	0.053	0.002	0.042	5.4	0.0000002
[SEX] + [GT] + [SEX × GT]	0.053	0.002	0.035	4.5	1.1
[SEX] + [GT] + [AGE] + [SEX × GT] + [SEX × AGE] + [GT × AGE] + [SEX × GT × AGE]	0.053	0.0008	0.013	1.6	6.7
[GT]	0.053	0.0007	0.012	1.5	0.0000008

GT – genotype, P(M) – prior model probability, P(M|data) – posterior model probabilities, BF_M – change from prior odds to posterior odds for each model, BF_{10} – Bayes factor.

Supplementary Table 14. Four-way Bayesian ANCOVA for the retrotransposon activity factored by sex, age, genotype, and experimental conditions

Models	P(M)	P(M|data)	BF_M	BF_{10}	error %
**SEX + AGE + GT + COND + ANCOVA + SEX×AGE + SEX×GT + AGE×GT + SEX×COND + AGE×COND + GT×COND + SEX×AGE×GT + SEX×AGE×COND + SEX×GT×COND + AGE×GT×COND + SEX×AGE×GT×COND**	**2.99e×10-3**	**0.56**	**417.27**	**1**	
SEX + AGE + GT + COND + ANCOVA + SEX×AGE + SEX×GT + AGE×GT + SEX×COND + AGE×COND + GT×COND + SEX×AGE×GT + SEX×GT×COND + AGE×GT×COND	2.99e×10-3	0.27	122.63	0.48	15.69
SEX + AGE + GT + COND + ANCOVA + SEX×AGE + SEX×GT + AGE×GT + SEX×COND + AGE×COND + GT×COND + SEX×AGE×GT + SEX×AGE×COND + SEX×GT×COND + AGE×GT×COND	2.99e×10-3	0.06	22.24	0.11	30.02
SEX + AGE + GT + COND + ANCOVA + SEX×AGE + SEX×GT + AGE×GT + SEX×COND + AGE×COND + GT×COND + SEX×GT×COND + AGE×GT×COND	2.99e×10-3	0.06	20.85	0.11	18.51
SEX + AGE + GT + COND + ANCOVA + SEX×AGE + SEX×GT + AGE×GT + SEX×COND + AGE×COND + GT×COND + SEX×AGE×GT + AGE×GT×COND	2.99e×10-3	0.03	9.31	0.05	26.91
SEX + AGE + GT + COND + ANCOVA + SEX×AGE + SEX×GT + AGE×GT + SEX×COND + AGE×COND + GT×COND + SEX×AGE×COND + SEX×GT×COND + AGE×GT×COND	2.99e×10-3	0.01	3.85	0.02	22.7
SEX + AGE + GT + COND + ANCOVA + SEX×AGE + SEX×GT + AGE×GT + SEX×COND + AGE×COND + GT×COND + AGE×GT×COND	2.99e×10-3	4.48e×10-3	1.5	8.05e×10-3	16.14
SEX + AGE + GT + COND + ANCOVA + SEX×AGE + AGE×GT + SEX×COND + AGE×COND + GT×COND + AGE×GT×COND	2.99e×10-3	3.37e×10-3	1.13	6.06e×10-3	15.96
SEX + AGE + GT + COND + ANCOVA + SEX×AGE + SEX×GT + AGE×GT + SEX×COND + AGE×COND + GT×COND + SEX×AGE×GT + SEX×AGE×COND + AGE×GT×COND	2.99e×10-3	3.34e×10-3	1.12	6.00e×10-3	18.67
SEX + AGE + GT + COND + ANCOVA + SEX×AGE + AGE×GT + SEX×COND + AGE×COND + GT×COND + SEX×AGE×COND + AGE×GT×COND	2.99e×10-3	1.19e×10-3	0.4	2.15e×10-3	49.22
SEX + AGE + GT + COND + ANCOVA + SEX×AGE + SEX×GT + AGE×GT + SEX×COND + AGE×COND + GT×COND + SEX×AGE×COND + AGE×GT×COND	2.99e×10-3	9.37e×10-4	0.31	1.69e×10-3	25
SEX + AGE + GT + COND + ANCOVA + SEX×AGE + SEX×GT + AGE×GT + SEX×COND + GT×COND + SEX×AGE×GT + SEX×GT×COND	2.99e×10-3	4.37e×10-4	0.15	7.86e×10-4	22.13
SEX + AGE + GT + COND + ANCOVA + SEX×AGE + SEX×GT + AGE×GT + SEX×COND + AGE×COND + GT×COND + SEX×AGE×GT + SEX×GT×COND	2.99e×10-3	3.88e×10-4	0.13	6.97e×10-4	17.78
SEX + AGE + GT + COND + ANCOVA + SEX×AGE + SEX×GT + AGE×GT + SEX×COND + SEX×AGE×GT	2.99e×10-3	1.26e×10-4	0.04	2.26e×10-4	15.29
SEX + AGE + GT + COND + ANCOVA + SEX×AGE + SEX×GT + AGE×GT + SEX×COND + AGE×COND + SEX×AGE×GT	2.99e×10-3	8.25e×10-5	0.03	1.48e×10-4	15.74
SEX + AGE + GT + COND + ANCOVA + SEX×AGE + SEX×GT + AGE×GT + SEX×COND + AGE×COND +	2.99e×10-3	6.26e×10-5	0.02	1.13e×10-4	19.56

GT×COND + SEX×AGE×GT + SEX×AGE×COND + SEX×GT×COND					
SEX + AGE + GT + COND + ANCOVA + SEX×AGE + SEX×GT + AGE×GT + SEX×COND + GT×COND + SEX×AGE×GT	2.99e×10-3	4.38e×10-5	0.01	7.88e×10-5	16.78
SEX + AGE + GT + COND + ANCOVA + SEX×AGE + SEX×GT + AGE×GT + SEX×COND + AGE×COND + GT×COND + SEX×AGE×GT	2.99e×10-3	2.91e×10-5	9.69e×10-3	5.23e×10-5	16.5
SEX + AGE + GT + COND + ANCOVA + SEX×AGE + SEX×GT + AGE×GT + SEX×COND + AGE×COND + GT×COND + SEX×GT×COND	2.99e×10-3	1.48e×10-5	4.92e×10-3	2.65e×10-5	39.86
SEX + AGE + GT + COND + ANCOVA + SEX×AGE + SEX×GT + AGE×GT + SEX×COND + AGE×COND + SEX×AGE×GT + SEX×AGE×COND	2.99e×10-3	1.20e×10-5	3.99e×10-3	2.15e×10-5	40.39
SEX + AGE + GT + COND + ANCOVA + SEX×AGE + SEX×GT + AGE×GT + SEX×COND	2.99e×10-3	1.15e×10-5	3.83e×10-3	2.07e×10-5	38.17
SEX + AGE + GT + COND + ANCOVA + SEX×AGE + SEX×GT + AGE×GT + SEX×COND + GT×COND + SEX×GT×COND	2.99e×10-3	9.02e×10-6	3.01e×10-3	1.62e×10-5	17.29
SEX + AGE + GT + COND + ANCOVA + SEX×AGE + AGE×GT + SEX×COND	2.99e×10-3	6.57e×10-6	2.19e×10-3	1.18e×10-5	14.29
SEX + AGE + GT + COND + ANCOVA + SEX×AGE + AGE×GT + SEX×COND + AGE×COND	2.99e×10-3	5.75e×10-6	1.92e×10-3	1.03e×10-5	33.62
SEX + AGE + GT + COND + ANCOVA + SEX×AGE + SEX×GT + AGE×GT + SEX×COND + AGE×COND	2.99e×10-3	5.11e×10-6	1.70e×10-3	9.18e×10-6	16.5
SEX + AGE + GT + COND + ANCOVA + SEX×AGE + SEX×GT + AGE×GT + SEX×COND + AGE×COND + GT×COND + SEX×AGE×GT + SEX×AGE×COND	2.99e×10-3	4.02e×10-6	1.34e×10-3	7.22e×10-6	33.45
SEX + AGE + GT + COND + ANCOVA + SEX×AGE + AGE×GT + SEX×COND + GT×COND	2.99e×10-3	2.30e×10-6	7.65e×10-4	4.13e×10-6	17.79
SEX + AGE + GT + COND + ANCOVA + SEX×AGE + AGE×GT + SEX×COND + AGE×COND + GT×COND	2.99e×10-3	1.86e×10-6	6.18e×10-4	3.34e×10-6	16.55
SEX + AGE + GT + COND + ANCOVA + SEX×AGE + SEX×GT + AGE×GT + SEX×COND + AGE×COND + GT×COND + SEX×AGE×COND + SEX×GT×COND	2.99e×10-3	1.71e×10-6	5.70e×10-4	3.08e×10-6	20.43
SEX + AGE + GT + COND + ANCOVA + SEX×AGE + SEX×GT + AGE×GT + SEX×COND + AGE×COND + GT×COND	2.99e×10-3	1.70e×10-6	5.66e×10-4	3.05e×10-6	16.4
SEX + AGE + GT + COND + ANCOVA + SEX×AGE + SEX×GT + AGE×GT + SEX×COND + GT×COND	2.99e×10-3	1.40e×10-6	4.65e×10-4	2.51e×10-6	15.56
SEX + AGE + GT + COND + ANCOVA + SEX×AGE + SEX×GT + AGE×GT + SEX×COND + AGE×COND + SEX×AGE×COND	2.99e×10-3	5.16e×10-7	1.72e×10-4	9.27e×10-7	16.06
SEX + AGE + GT + COND + ANCOVA + SEX×AGE + AGE×GT + SEX×COND + AGE×COND + SEX×AGE×COND	2.99e×10-3	4.46e×10-7	1.49e×10-4	8.02e×10-7	14.68
SEX + AGE + GT + COND + ANCOVA + SEX×AGE + AGE×GT + SEX×COND + AGE×COND + GT×COND + SEX×AGE×COND	2.99e×10-3	3.07e×10-7	1.02e×10-4	5.52e×10-7	26.69
SEX + AGE + GT + COND + ANCOVA + SEX×AGE + SEX×GT + AGE×GT + SEX×COND + AGE×COND + GT×COND + SEX×AGE×COND	2.99e×10-3	1.55e×10-7	5.16e×10-5	2.79e×10-7	20.29

SEX + AGE + GT + COND + ANCOVA + SEX×AGE + SEX×GT + AGE×GT + AGE×COND + GT×COND + SEX×AGE×GT + AGE×GT×COND	2.99e×10-3	2.28e×10-10	7.59e×10-8	4.10e×10-10	25.62
SEX + AGE + GT + COND + ANCOVA + SEX×AGE + SEX×GT + AGE×GT + AGE×COND + GT×COND + AGE×GT×COND	2.99e×10-3	4.05e×10-11	1.35e×10-8	7.29e×10-11	18.99
SEX + AGE + GT + COND + ANCOVA + SEX×AGE + SEX×GT + AGE×GT + GT×COND + SEX×AGE×GT	2.99e×10-3	2.23e×10-11	7.44e×10-9	4.02e×10-11	66.37
SEX + AGE + GT + COND + ANCOVA + SEX×AGE + SEX×GT + AGE×GT + SEX×AGE×GT	2.99e×10-3	1.82e×10-11	6.07e×10-9	3.28e×10-11	15.1
SEX + AGE + GT + COND + ANCOVA + SEX×AGE + SEX×GT + AGE×GT + AGE×COND + SEX×AGE×GT	2.99e×10-3	8.29e×10-12	2.76e×10-9	1.49e×10-11	15.5
SEX + AGE + GT + COND + ANCOVA + SEX×AGE + SEX×GT + AGE×GT + AGE×COND + GT×COND + SEX×AGE×GT	2.99e×10-3	1.63e×10-12	5.43e×10-10	2.93e×10-12	17.12
SEX + AGE + GT + COND + ANCOVA + SEX×AGE + AGE×GT + AGE×COND + GT×COND + AGE×GT×COND	2.99e×10-3	1.34e×10-12	4.45e×10-10	2.40e×10-12	17.46
SEX + AGE + GT + COND + ANCOVA + SEX×AGE + SEX×GT + AGE×GT	2.99e×10-3	9.78e×10-13	3.26e×10-10	1.76e×10-12	14.4
SEX + AGE + GT + COND + ANCOVA + SEX×AGE + SEX×GT + AGE×GT + AGE×COND	2.99e×10-3	7.70e×10-13	2.56e×10-10	1.38e×10-12	24.96
SEX + AGE + GT + COND + ANCOVA + SEX×GT + AGE×GT + SEX×COND + AGE×COND + GT×COND + SEX×GT×COND + AGE×GT×COND	2.99e×10-3	7.10e×10-13	2.36e×10-10	1.28e×10-12	23.36
SEX + AGE + GT + COND + ANCOVA + AGE×GT + SEX×COND + AGE×COND + GT×COND + AGE×GT×COND	2.99e×10-3	1.51e×10-13	5.04e×10-11	2.72e×10-13	16.1
SEX + AGE + GT + COND + ANCOVA + SEX×AGE + SEX×GT + AGE×GT + GT×COND	2.99e×10-3	7.46e×10-14	2.48e×10-11	1.34e×10-13	14.58
SEX + AGE + GT + COND + ANCOVA + SEX×GT + AGE×GT + SEX×COND + AGE×COND + GT×COND + AGE×GT×COND	2.99e×10-3	6.19e×10-14	2.06e×10-11	1.11e×10-13	19.08
SEX + AGE + GT + COND + ANCOVA + SEX×AGE + SEX×GT + AGE×GT + AGE×COND + GT×COND	2.99e×10-3	6.11e×10-14	2.03e×10-11	1.10e×10-13	17.19
SEX + AGE + GT + COND + ANCOVA + SEX×AGE + AGE×GT	2.99e×10-3	5.10e×10-14	1.70e×10-11	9.17e×10-14	14.34
SEX + AGE + GT + COND + ANCOVA + SEX×AGE + AGE×GT + AGE×COND	2.99e×10-3	3.36e×10-14	1.12e×10-11	6.04e×10-14	17.19
SEX + AGE + GT + COND + ANCOVA + SEX×AGE + AGE×GT + GT×COND	2.99e×10-3	7.36e×10-15	2.45e×10-12	1.32e×10-14	16.17
SEX + AGE + GT + COND + ANCOVA + SEX×AGE + AGE×GT + AGE×COND + GT×COND	2.99e×10-3	4.60e×10-15	1.53e×10-12	8.27e×10-15	14.71
SEX + AGE + COND + ANCOVA + SEX×AGE + SEX×COND + AGE×COND	2.99e×10-3	3.84e×10-17	1.28e×10-14	6.90e×10-17	18.4
SEX + AGE + GT + COND + ANCOVA + AGE×GT + SEX×COND + AGE×COND	2.99e×10-3	1.97e×10-17	6.55e×10-15	3.54e×10-17	26.23
SEX + AGE + GT + COND + ANCOVA + SEX×GT + AGE×GT + SEX×COND + AGE×COND + GT×COND + SEX×GT×COND	2.99e×10-3	1.64e×10-17	5.46e×10-15	2.95e×10-17	32.1
SEX + AGE + GT + COND + ANCOVA + SEX×GT + AGE×GT + SEX×COND + AGE×COND	2.99e×10-3	8.80e×10-18	2.93e×10-15	1.58e×10-17	15.84

SEX + AGE + GT + COND + ANCOVA + AGE×GT + SEX×COND + AGE×COND + GT×COND	2.99e×10-3	8.19e×10-18	2.73e×10-15	1.47e×10-17	15.28
SEX + AGE + COND + ANCOVA + SEX×AGE + SEX×COND + AGE×COND + SEX×AGE×COND	2.99e×10-3	6.16e×10-18	2.05e×10-15	1.11e×10-17	15.71
SEX + AGE + GT + COND + ANCOVA + SEX×AGE + SEX×COND + AGE×COND	2.99e×10-3	5.68e×10-18	1.89e×10-15	1.02e×10-17	14.78
SEX + AGE + GT + COND + ANCOVA + SEX×AGE + SEX×GT + SEX×COND + AGE×COND	2.99e×10-3	4.77e×10-18	1.59e×10-15	8.58e×10-18	15.37
SEX + AGE + GT + COND + ANCOVA + AGE×GT + SEX×COND	2.99e×10-3	2.91e×10-18	9.68e×10-16	5.23e×10-18	15.76
SEX + AGE + GT + COND + ANCOVA + SEX×GT + AGE×GT + SEX×COND + AGE×COND + GT×COND	2.99e×10-3	1.72e×10-18	5.73e×10-16	3.09e×10-18	15.69
SEX + AGE + GT + COND + ANCOVA + SEX×GT + AGE×GT + SEX×COND + GT×COND + SEX×GT×COND	2.99e×10-3	1.65e×10-18	5.50e×10-16	2.97e×10-18	29.22
SEX + AGE + GT + COND + ANCOVA + SEX×AGE + SEX×GT + SEX×COND + AGE×COND + GT×COND + SEX×GT×COND	2.99e×10-3	1.56e×10-18	5.20e×10-16	2.81e×10-18	22.99
SEX + AGE + COND + ANCOVA + SEX×AGE + SEX×COND	2.99e×10-3	1.48e×10-18	4.93e×10-16	2.66e×10-18	14.81
SEX + AGE + GT + COND + ANCOVA + SEX×AGE + SEX×GT + SEX×COND + AGE×COND + GT×COND	2.99e×10-3	1.48e×10-18	4.92e×10-16	2.66e×10-18	56.81
SEX + AGE + GT + COND + ANCOVA + SEX×AGE + SEX×COND + AGE×COND + GT×COND	2.99e×10-3	1.23e×10-18	4.11e×10-16	2.22e×10-18	18.2
SEX + AGE + GT + COND + ANCOVA + SEX×GT + AGE×GT + SEX×COND	2.99e×10-3	1.19e×10-18	3.97e×10-16	2.15e×10-18	17.13
SEX + AGE + GT + COND + ANCOVA + SEX×AGE + SEX×COND + AGE×COND + SEX×AGE×COND	2.99e×10-3	9.96e×10-19	3.32e×10-16	1.79e×10-18	14.89
SEX + AGE + GT + COND + ANCOVA + AGE×GT + SEX×COND + GT×COND	2.99e×10-3	8.70e×10-19	2.90e×10-16	1.56e×10-18	17.13
SEX + AGE + GT + COND + ANCOVA + SEX×AGE + SEX×GT + SEX×COND + AGE×COND + SEX×AGE×COND	2.99e×10-3	6.23e×10-19	2.08e×10-16	1.12e×10-18	18.78
SEX + AGE + GT + COND + ANCOVA + SEX×GT + AGE×GT + SEX×COND + GT×COND	2.99e×10-3	3.55e×10-19	1.18e×10-16	6.38e×10-19	37.81
SEX + AGE + GT + COND + ANCOVA + SEX×AGE + SEX×COND	2.99e×10-3	2.41e×10-19	8.04e×10-17	4.34e×10-19	15.01
SEX + AGE + GT + COND + ANCOVA + SEX×AGE + SEX×COND + AGE×COND + GT×COND + SEX×AGE×COND	2.99e×10-3	1.93e×10-19	6.41e×10-17	3.46e×10-19	22.71
SEX + AGE + GT + COND + ANCOVA + SEX×AGE + SEX×GT + SEX×COND + AGE×COND + GT×COND + SEX×AGE×COND + SEX×GT×COND	2.99e×10-3	1.64e×10-19	5.45e×10-17	2.94e×10-19	15.04
SEX + AGE + GT + COND + ANCOVA + SEX×AGE + SEX×GT + SEX×COND	2.99e×10-3	1.50e×10-19	4.99e×10-17	2.69e×10-19	15.83
SEX + AGE + GT + COND + ANCOVA + SEX×AGE + SEX×GT + SEX×COND + AGE×COND + GT×COND + SEX×AGE×COND	2.99e×10-3	1.03e×10-19	3.42e×10-17	1.84e×10-19	16.24
SEX + AGE + GT + COND + ANCOVA + SEX×AGE + SEX×COND + GT×COND	2.99e×10-3	3.40e×10-20	1.13e×10-17	6.11e×10-20	14.81
SEX + AGE + GT + COND + ANCOVA + SEX×AGE + SEX×GT + SEX×COND + GT×COND + SEX×GT×COND	2.99e×10-3	1.97e×10-20	6.56e×10-18	3.54e×10-20	15.01

SEX + AGE + GT + COND + ANCOVA + SEX×AGE + SEX×GT + SEX×COND + GT×COND	2.99e×10-3	1.60e×10-20	5.33e×10-18	2.88e×10-20	16.57
SEX + AGE + GT + COND + ANCOVA + SEX×GT + AGE×GT + AGE×COND + GT×COND + AGE×GT×COND	2.99e×10-3	1.17e×10-20	3.91e×10-18	2.11e×10-20	14.95
SEX + AGE + GT + COND + ANCOVA + AGE×GT + AGE×COND + GT×COND + AGE×GT×COND	2.99e×10-3	1.90e×10-21	6.34e×10-19	3.42e×10-21	18.46
SEX + AGE + GT + COND + ANCOVA + SEX×GT + AGE×GT + AGE×COND	2.99e×10-3	5.59e×10-23	1.86e×10-20	1.01e×10-22	66.59
SEX + AGE + GT + COND + ANCOVA + AGE×GT + AGE×COND	2.99e×10-3	7.99e×10-24	2.66e×10-21	1.44e×10-23	14.56
SEX + AGE + GT + COND + ANCOVA + SEX×GT + AGE×GT	2.99e×10-3	5.42e×10-24	1.80e×10-21	9.74e×10-24	24.53
SEX + AGE + GT + COND + ANCOVA + SEX×GT + AGE×GT + AGE×COND + GT×COND	2.99e×10-3	2.50e×10-24	8.34e×10-22	4.50e×10-24	18.13
SEX + AGE + GT + COND + ANCOVA + AGE×GT	2.99e×10-3	1.49e×10-24	4.95e×10-22	2.67e×10-24	14.66
SEX + AGE + GT + COND + ANCOVA + AGE×GT + AGE×COND + GT×COND	2.99e×10-3	1.29e×10-24	4.30e×10-22	2.32e×10-24	14.91
SEX + AGE + GT + COND + ANCOVA + SEX×AGE + SEX×GT + AGE×COND	2.99e×10-3	1.12e×10-24	3.72e×10-22	2.01e×10-24	14.96
SEX + AGE + GT + COND + ANCOVA + SEX×GT + AGE×GT + GT×COND	2.99e×10-3	5.01e×10-25	1.67e×10-22	9.01e×10-25	18.44
SEX + AGE + COND + ANCOVA + SEX×AGE + AGE×COND	2.99e×10-3	4.22e×10-25	1.41e×10-22	7.60e×10-25	16.21
SEX + AGE + GT + COND + ANCOVA + AGE×GT + GT×COND	2.99e×10-3	2.36e×10-25	7.85e×10-23	4.24e×10-25	20.12
SEX + AGE + GT + COND + ANCOVA + SEX×AGE + AGE×COND	2.99e×10-3	1.48e×10-25	4.91e×10-23	2.65e×10-25	14.42
SEX + AGE + GT + ANCOVA + SEX×AGE + SEX×GT + AGE×GT + SEX×AGE×GT	2.99e×10-3	9.83e×10-26	3.27e×10-23	1.77e×10-25	14.22
SEX + AGE + COND + ANCOVA + SEX×COND + AGE×COND	2.99e×10-3	8.03e×10-26	2.67e×10-23	1.44e×10-25	24.87
SEX + AGE + GT + COND + ANCOVA + SEX×AGE + SEX×GT + AGE×COND + GT×COND	2.99e×10-3	7.09e×10-26	2.36e×10-23	1.27e×10-25	15.67
SEX + AGE + GT + COND + ANCOVA + SEX×AGE + SEX×GT	2.99e×10-3	4.65e×10-26	1.55e×10-23	8.36e×10-26	14.4
SEX + AGE + GT + COND + ANCOVA + SEX×AGE + AGE×COND + GT×COND	2.99e×10-3	1.85e×10-26	6.14e×10-24	3.32e×10-26	24.67
SEX + AGE + COND + ANCOVA + SEX×AGE	2.99e×10-3	1.69e×10-26	5.63e×10-24	3.04e×10-26	14.01
SEX + AGE + GT + ANCOVA + SEX×AGE + SEX×GT + AGE×GT	2.99e×10-3	8.09e×10-27	2.69e×10-24	1.45e×10-26	14.42
SEX + AGE + GT + COND + ANCOVA + SEX×COND + AGE×COND	2.99e×10-3	6.93e×10-27	2.31e×10-24	1.25e×10-26	14.35
SEX + AGE + GT + COND + ANCOVA + SEX×AGE	2.99e×10-3	6.31e×10-27	2.10e×10-24	1.13e×10-26	14.46
SEX + AGE + GT + COND + ANCOVA + SEX×AGE + SEX×GT + GT×COND	2.99e×10-3	2.99e×10-27	9.97e×10-25	5.38e×10-27	15.36
SEX + AGE + GT + COND + ANCOVA + SEX×GT + SEX×COND + AGE×COND	2.99e×10-3	1.88e×10-27	6.25e×10-25	3.37e×10-27	17.79

SEX + AGE + GT + COND + ANCOVA + SEX×COND + AGE×COND + GT×COND	2.99e×10-3	1.31e×10-27	4.36e×10-25	2.36e×10-27	16.07
SEX + AGE + GT + COND + ANCOVA + SEX×GT + SEX×COND + AGE×COND + GT×COND + SEX×GT×COND	2.99e×10-3	6.53e×10-28	2.18e×10-25	1.17e×10-27	15.13
SEX + AGE + GT + COND + ANCOVA + SEX×AGE + GT×COND	2.99e×10-3	6.21e×10-28	2.07e×10-25	1.12e×10-27	14.22
SEX + AGE + COND + ANCOVA + SEX×COND	2.99e×10-3	2.32e×10-28	7.71e×10-26	4.16e×10-28	13.89
SEX + AGE + GT + COND + ANCOVA + SEX×GT + SEX×COND + AGE×COND + GT×COND	2.99e×10-3	2.31e×10-28	7.70e×10-26	4.16e×10-28	15.2
SEX + AGE + GT + ANCOVA + SEX×AGE + AGE×GT	2.99e×10-3	7.08e×10-29	2.36e×10-26	1.27e×10-28	16.87
SEX + COND + ANCOVA + SEX×COND	2.99e×10-3	6.86e×10-29	2.28e×10-26	1.23e×10-28	13.71
SEX + AGE + GT + COND + ANCOVA + SEX×COND	2.99e×10-3	2.11e×10-29	7.03e×10-27	3.80e×10-29	14.06
SEX + GT + COND + ANCOVA + SEX×COND	2.99e×10-3	5.80e×10-30	1.93e×10-27	1.04e×10-29	14.8
SEX + AGE + GT + COND + ANCOVA + SEX×GT + SEX×COND	2.99e×10-3	4.93e×10-30	1.64e×10-27	8.87e×10-30	14.91
SEX + AGE + GT + COND + ANCOVA + SEX×COND + GT×COND	2.99e×10-3	2.84e×10-30	9.45e×10-28	5.10e×10-30	15.12
SEX + GT + COND + ANCOVA + SEX×GT + SEX×COND	2.99e×10-3	1.14e×10-30	3.80e×10-28	2.05e×10-30	16.09
SEX + AGE + GT + COND + ANCOVA + SEX×GT + SEX×COND + GT×COND + SEX×GT×COND	2.99e×10-3	8.92e×10-31	2.97e×10-28	1.60e×10-30	15.23
SEX + GT + COND + ANCOVA + SEX×COND + GT×COND	2.99e×10-3	6.86e×10-31	2.28e×10-28	1.23e×10-30	14.48
SEX + AGE + GT + COND + ANCOVA + SEX×GT + SEX×COND + GT×COND	2.99e×10-3	6.38e×10-31	2.12e×10-28	1.15e×10-30	18.82
SEX + GT + COND + ANCOVA + SEX×GT + SEX×COND + GT×COND + SEX×GT×COND	2.99e×10-3	1.37e×10-31	4.55e×10-29	2.46e×10-31	15.01
SEX + GT + COND + ANCOVA + SEX×GT + SEX×COND + GT×COND	2.99e×10-3	1.23e×10-31	4.11e×10-29	2.22e×10-31	14.45
SEX + AGE + COND + ANCOVA + AGE×COND	2.99e×10-3	2.32e×10-32	7.74e×10-30	4.18e×10-32	14.18
SEX + AGE + GT + COND + ANCOVA + SEX×GT + AGE×COND	2.99e×10-3	8.28e×10-33	2.76e×10-30	1.49e×10-32	14.45
SEX + AGE + GT + COND + ANCOVA + AGE×COND	2.99e×10-3	4.34e×10-33	1.45e×10-30	7.81e×10-33	14.16
SEX + AGE + GT + COND + ANCOVA + SEX×GT + AGE×COND + GT×COND	2.99e×10-3	6.97e×10-34	2.32e×10-31	1.25e×10-33	15.44
SEX + AGE + GT + COND + ANCOVA + AGE×COND + GT×COND	2.99e×10-3	4.43e×10-34	1.47e×10-31	7.96e×10-34	14.4
SEX + COND + ANCOVA	2.99e×10-3	1.56e×10-34	5.18e×10-32	2.80e×10-34	13.78
SEX + AGE + COND + ANCOVA	2.99e×10-3	1.30e×10-34	4.33e×10-32	2.34e×10-34	13.76
SEX + AGE + GT + COND + ANCOVA + SEX×GT	2.99e×10-3	2.98e×10-35	9.91e×10-33	5.35e×10-35	13.96
SEX + GT + COND + ANCOVA	2.99e×10-3	1.89e×10-35	6.30e×10-33	3.40e×10-35	14.69
SEX + GT + COND + ANCOVA + SEX×GT	2.99e×10-3	1.84e×10-35	6.13e×10-33	3.31e×10-35	19.19
SEX + AGE + GT + COND + ANCOVA	2.99e×10-3	1.84e×10-35	6.13e×10-33	3.31e×10-35	14.93
SEX + AGE + GT + ANCOVA + SEX×GT + AGE×GT	2.99e×10-3	8.12e×10-36	2.70e×10-33	1.46e×10-35	15.33

SEX + AGE + GT + COND + ANCOVA + SEX×GT + GT×COND	2.99e×10-3	3.18e×10-36	1.06e×10-33	5.71e×10-36	14.46
SEX + AGE + GT + COND + ANCOVA + GT×COND	2.99e×10-3	2.10e×10-36	6.98e×10-34	3.77e×10-36	14.37
SEX + GT + COND + ANCOVA + GT×COND	2.99e×10-3	1.90e×10-36	6.34e×10-34	3.42e×10-36	15.77
SEX + GT + COND + ANCOVA + SEX×GT + GT×COND	2.99e×10-3	1.86e×10-36	6.20e×10-34	3.35e×10-36	17.59
SEX + AGE + GT + ANCOVA + AGE×GT	2.99e×10-3	4.97e×10-37	1.66e×10-34	8.94e×10-37	14.14
SEX + AGE + GT + ANCOVA + SEX×AGE + SEX×GT	2.99e×10-3	1.35e×10-39	4.50e×10-37	2.43e×10-39	14.37
SEX + AGE + GT + ANCOVA + SEX×AGE	2.99e×10-3	3.43e×10-41	1.14e×10-38	6.16e×10-41	13.96
SEX + AGE + ANCOVA + SEX×AGE	2.99e×10-3	2.73e×10-42	9.10e×10-40	4.91e×10-42	14.43
SEX + GT + ANCOVA + SEX×GT	2.99e×10-3	2.19e×10-46	7.29e×10-44	3.93e×10-46	13.83
SEX + AGE + GT + ANCOVA + SEX×GT	2.99e×10-3	1.18e×10-46	3.93e×10-44	2.12e×10-46	13.98
SEX + GT + ANCOVA	2.99e×10-3	5.14e×10-47	1.71e×10-44	9.23e×10-47	13.69
SEX + ANCOVA	2.99e×10-3	2.88e×10-47	9.60e×10-45	5.18e×10-47	13.59
SEX + AGE + GT + ANCOVA	2.99e×10-3	2.56e×10-47	8.51e×10-45	4.60e×10-47	42.22
SEX + AGE + ANCOVA	2.99e×10-3	6.29e×10-48	2.09e×10-45	1.13e×10-47	15.32
AGE + GT + COND + ANCOVA + AGE×GT + AGE×COND + GT×COND + AGE×GT×COND	2.99e×10-3	6.33e×10-186	2.11e×10-183	1.14e×10-185	14.6
AGE + GT + COND + ANCOVA + AGE×GT + GT×COND	2.99e×10-3	3.15e×10-186	1.05e×10-183	5.66e×10-186	13.97
COND + ANCOVA	2.99e×10-3	1.86e×10-186	6.18e×10-184	3.34e×10-186	13.49
AGE + GT + COND + ANCOVA + AGE×GT	2.99e×10-3	8.53e×10-187	2.84e×10-184	1.53e×10-186	13.96
AGE + COND + ANCOVA	2.99e×10-3	3.58e×10-187	1.19e×10-184	6.44e×10-187	13.53
GT + COND + ANCOVA + GT×COND	2.99e×10-3	3.20e×10-187	1.06e×10-184	5.75e×10-187	13.59
AGE + GT + COND + ANCOVA + AGE×GT + AGE×COND + GT×COND	2.99e×10-3	3.09e×10-187	1.03e×10-184	5.55e×10-187	14.83
GT + COND + ANCOVA	2.99e×10-3	1.98e×10-187	6.60e×10-185	3.56e×10-187	13.54
AGE + GT + COND + ANCOVA + AGE×GT + AGE×COND	2.99e×10-3	7.77e×10-188	2.59e×10-185	1.40e×10-187	14.01
AGE + GT + COND + ANCOVA + GT×COND	2.99e×10-3	5.97e×10-188	1.99e×10-185	1.07e×10-187	13.79
AGE + COND + ANCOVA + AGE×COND	2.99e×10-3	4.56e×10-188	1.52e×10-185	8.19e×10-188	13.68
AGE + GT + COND + ANCOVA	2.99e×10-3	3.36e×10-188	1.12e×10-185	6.04e×10-188	13.71
AGE + GT + COND + ANCOVA + AGE×COND + GT×COND	2.99e×10-3	9.14e×10-189	3.04e×10-186	1.64e×10-188	14.03
AGE + GT + COND + ANCOVA + AGE×COND	2.99e×10-3	4.34e×10-189	1.45e×10-186	7.81e×10-189	14.15

ANCOVA	2.99e×10-3	1.89e×10-201	6.30e×10-199	3.40e×10-201	13.43
AGE + GT + ANCOVA + AGE×GT	2.99e×10-3	1.83e×10-202	6.08e×10-200	3.28e×10-202	14.01
AGE + ANCOVA	2.99e×10-3	1.53e×10-202	5.09e×10-200	2.75e×10-202	13.67
GT + ANCOVA	2.99e×10-3	1.39e×10-202	4.62e×10-200	2.49e×10-202	13.51
AGE + GT + ANCOVA	2.99e×10-3	1.15e×10-203	3.82e×10-201	2.06e×10-203	13.62
SEX + AGE + GT + COND + SEX×AGE + AGE×GT + SEX×COND	2.99e×10-3	0	0	0	13.81
SEX + AGE + COND + SEX×AGE + SEX×COND	2.99e×10-3	0	0	0	14.14
SEX + AGE + GT + COND + SEX×AGE + SEX×GT + AGE×GT + SEX×COND	2.99e×10-3	0	0	0	14.01
SEX + AGE + GT + COND + SEX×AGE + AGE×GT + SEX×COND + AGE×COND	2.99e×10-3	0	0	0	14.28
SEX + AGE + GT + COND + SEX×AGE + AGE×GT + SEX×COND + GT×COND	2.99e×10-3	0	0	0	14.08
SEX + COND + SEX×COND	2.99e×10-3	0	0	0	13.49
SEX + AGE + GT + COND + SEX×AGE + AGE×GT	2.99e×10-3	0	0	0	14.21
SEX + AGE + COND + SEX×AGE + SEX×COND + AGE×COND	2.99e×10-3	0	0	0	13.68
SEX + AGE + GT + COND + SEX×AGE + SEX×GT + AGE×GT + SEX×COND + SEX×AGE×GT	2.99e×10-3	0	0	0	14.58
SEX + AGE + COND + SEX×AGE	2.99e×10-3	0	0	0	14.07
SEX + COND	2.99e×10-3	0	0	0	13.46
SEX + AGE + GT + COND + SEX×AGE + SEX×COND	2.99e×10-3	0	0	0	14.15
SEX + AGE + GT + COND + SEX×AGE + SEX×GT + AGE×GT	2.99e×10-3	0	0	0	13.83
SEX + AGE + GT + COND + AGE×GT + SEX×COND	2.99e×10-3	0	0	0	19.54
SEX + AGE + GT + COND + SEX×AGE + SEX×GT + AGE×GT + SEX×COND + AGE×COND	2.99e×10-3	0	0	0	18.03
SEX + AGE + GT + COND + SEX×AGE + AGE×GT + SEX×COND + AGE×COND + GT×COND	2.99e×10-3	0	0	0	14.1
SEX + AGE + GT + COND + SEX×AGE + SEX×GT + AGE×GT + SEX×COND + GT×COND	2.99e×10-3	0	0	0	14.04
SEX + AGE + COND + SEX×COND	2.99e×10-3	0	0	0	13.78
SEX + AGE + GT + COND + SEX×AGE + AGE×GT + SEX×COND + AGE×COND + SEX×AGE×COND	2.99e×10-3	0	0	0	14.03
SEX + AGE + COND + SEX×AGE + SEX×COND + AGE×COND + SEX×AGE×COND	2.99e×10-3	0	0	0	15.92
SEX + AGE + GT + COND + SEX×AGE + AGE×GT + AGE×COND	2.99e×10-3	0	0	0	13.91
SEX + AGE + GT + COND + SEX×AGE + AGE×GT + SEX×COND + AGE×COND + GT×COND + AGE×GT×COND	2.99e×10-3	0	0	0	16.51

SEX + AGE + GT + COND + SEX×AGE + AGE×GT + GT×COND	2.99e×10-3	0	0	0	14.31
SEX + AGE + GT + COND + SEX×AGE + SEX×GT + AGE×GT + SEX×AGE×GT	2.99e×10-3	0	0	0	18.54
SEX + AGE + COND + SEX×AGE + AGE×COND	2.99e×10-3	0	0	0	13.61
SEX + AGE + GT + COND + SEX×AGE + SEX×COND + AGE×COND	2.99e×10-3	0	0	0	17.14
SEX + GT + COND + SEX×COND	2.99e×10-3	0	0	0	13.84
SEX + AGE + COND + SEX×COND + AGE×COND	2.99e×10-3	0	0	0	13.79
SEX + AGE + GT + COND + SEX×AGE + SEX×GT + AGE×GT + SEX×COND + AGE×COND + SEX×AGE×GT	2.99e×10-3	0	0	0	14.48
SEX + AGE + GT + COND + SEX×AGE + SEX×GT + AGE×GT + SEX×COND + GT×COND + SEX×AGE×GT	2.99e×10-3	0	0	0	15.11
SEX + AGE + GT + COND + SEX×AGE + SEX×GT + AGE×GT + SEX×COND + GT×COND + SEX×GT×COND	2.99e×10-3	0	0	0	14.85
SEX + AGE + GT + COND + AGE×GT + SEX×COND + AGE×COND	2.99e×10-3	0	0	0	17.03
SEX + AGE + GT + COND + AGE×GT	2.99e×10-3	0	0	0	19.7
SEX + AGE + GT + COND + SEX×AGE + SEX×GT + AGE×GT + AGE×COND	2.99e×10-3	0	0	0	21.64
SEX + AGE + GT + COND + SEX×AGE + SEX×GT + SEX×COND	2.99e×10-3	0	0	0	14.5
SEX + AGE + GT + COND + SEX×AGE + SEX×COND + GT×COND	2.99e×10-3	0	0	0	13.95
SEX + AGE + COND	2.99e×10-3	0	0	0	13.5
SEX + AGE + GT + COND + SEX×AGE	2.99e×10-3	0	0	0	14.09
SEX + AGE + GT + COND + SEX×AGE + SEX×GT + AGE×GT + GT×COND	2.99e×10-3	0	0	0	17.99
SEX + AGE + GT + COND + SEX×GT + AGE×GT + SEX×COND	2.99e×10-3	0	0	0	14.63
SEX + AGE + GT + COND + SEX×AGE + SEX×GT + AGE×GT + SEX×COND + AGE×COND + SEX×AGE×COND	2.99e×10-3	0	0	0	14.8
SEX + GT + COND	2.99e×10-3	0	0	0	13.5
SEX + AGE + GT + COND + SEX×AGE + SEX×GT + AGE×GT + SEX×COND + AGE×COND + GT×COND	2.99e×10-3	0	0	0	14.81
SEX + AGE + GT + COND + AGE×GT + SEX×COND + GT×COND	2.99e×10-3	0	0	0	13.89
SEX + AGE + GT + COND + SEX×AGE + SEX×GT + AGE×GT + SEX×COND + AGE×COND + GT×COND + AGE×GT×COND	2.99e×10-3	0	0	0	15.43
SEX + AGE + GT + COND + SEX×AGE + AGE×GT + SEX×COND + AGE×COND + GT×COND + SEX×AGE×COND	2.99e×10-3	0	0	0	15.26
SEX + AGE + GT + COND + SEX×AGE + SEX×GT + AGE×GT + SEX×COND + GT×COND + SEX×AGE×GT + SEX×GT×COND	2.99e×10-3	0	0	0	16.94
SEX + AGE + COND + AGE×COND	2.99e×10-3	0	0	0	19.7

SEX + AGE + GT + COND + SEX×AGE + AGE×GT + SEX×COND + AGE×COND + GT×COND + SEX×AGE×COND + AGE×GT×COND	2.99e×10-3	0	0	0	20.35
SEX + AGE + GT + COND + SEX×AGE + AGE×GT + AGE×COND + GT×COND	2.99e×10-3	0	0	0	14.28
SEX + AGE + GT + COND + SEX×COND	2.99e×10-3	0	0	0	13.81
SEX + AGE + GT + COND + SEX×AGE + SEX×GT + SEX×COND + AGE×COND	2.99e×10-3	0	0	0	15.52
SEX + AGE + GT + COND + SEX×AGE + SEX×GT + AGE×GT + AGE×COND + SEX×AGE×GT	2.99e×10-3	0	0	0	14.47
SEX + AGE + GT + COND + SEX×AGE + SEX×COND + AGE×COND + SEX×AGE×COND	2.99e×10-3	0	0	0	15.32
SEX + AGE + GT + COND + SEX×AGE + SEX×COND + AGE×COND + GT×COND	2.99e×10-3	0	0	0	14.1
SEX + AGE + GT + COND + SEX×GT + AGE×GT	2.99e×10-3	0	0	0	13.73
SEX + GT + COND + SEX×GT + SEX×COND	2.99e×10-3	0	0	0	14.66
SEX + AGE + GT + COND + SEX×AGE + SEX×GT + AGE×GT + GT×COND + SEX×AGE×GT	2.99e×10-3	0	0	0	14.51
SEX + AGE + GT + COND + AGE×GT + AGE×COND	2.99e×10-3	0	0	0	14.03
SEX + AGE + GT + COND + SEX×AGE + AGE×COND	2.99e×10-3	0	0	0	13.88
SEX + GT + COND + SEX×COND + GT×COND	2.99e×10-3	0	0	0	13.76
SEX + AGE + GT + COND + SEX×AGE + SEX×GT	2.99e×10-3	0	0	0	14.43
SEX + AGE + GT + COND + SEX×AGE + SEX×GT + AGE×GT + SEX×COND + AGE×COND + SEX×AGE×GT + SEX×AGE×COND	2.99e×10-3	0	0	0	17.17
SEX + AGE + GT + COND + SEX×AGE + SEX×GT + AGE×GT + SEX×COND + AGE×COND + GT×COND + SEX×AGE×GT	2.99e×10-3	0	0	0	15
SEX + AGE + GT + COND + SEX×AGE + AGE×GT + AGE×COND + GT×COND + AGE×GT×COND	2.99e×10-3	0	0	0	17.58
SEX + AGE + GT + COND + SEX×COND + AGE×COND	2.99e×10-3	0	0	0	13.77
SEX + AGE + GT + COND + SEX×AGE + SEX×GT + AGE×GT + SEX×COND + AGE×COND + GT×COND + SEX×GT×COND	2.99e×10-3	0	0	0	14.42
SEX + AGE + GT + COND + SEX×GT + AGE×GT + SEX×COND + AGE×COND	2.99e×10-3	0	0	0	16.59
SEX + AGE + GT + COND + AGE×GT + GT×COND	2.99e×10-3	0	0	0	31.92
SEX + AGE + GT + COND + AGE×GT + SEX×COND + AGE×COND + GT×COND	2.99e×10-3	0	0	0	14.2
SEX + AGE + GT + COND + AGE×GT + SEX×COND + AGE×COND + GT×COND + AGE×GT×COND	2.99e×10-3	0	0	0	14.07
SEX + AGE + GT + COND + SEX×AGE + SEX×GT + AGE×GT + SEX×COND + AGE×COND + GT×COND + SEX×GT×COND + AGE×GT×COND	2.99e×10-3	0	0	0	15.03
SEX + AGE + GT + COND + SEX×AGE + SEX×GT + AGE×GT + SEX×COND + AGE×COND + GT×COND + SEX×AGE×GT + AGE×GT×COND	2.99e×10-3	0	0	0	14.76

SEX + AGE + GT + COND + SEX×AGE + SEX×GT + SEX×COND + GT×COND	2.99e×10-3	0	0	0	20.02
SEX + AGE + GT + COND + SEX×AGE + SEX×GT + AGE×GT + SEX×COND + AGE×COND + GT×COND + SEX×AGE×GT + SEX×GT×COND	2.99e×10-3	0	0	0	36.75
SEX + AGE + GT + COND + SEX×AGE + SEX×GT + AGE×GT + AGE×COND + GT×COND	2.99e×10-3	0	0	0	14.31
SEX + GT + COND + SEX×GT	2.99e×10-3	0	0	0	13.73
SEX + AGE + GT + SEX×AGE + AGE×GT	2.99e×10-3	0	0	0	13.63
SEX + AGE + GT + COND + SEX×GT + AGE×GT + GT×COND	2.99e×10-3	0	0	0	78.36
SEX + AGE + GT + COND + SEX×AGE + GT×COND	2.99e×10-3	0	0	0	13.76
SEX + AGE + GT + COND + SEX×AGE + SEX×GT + AGE×GT + SEX×COND + AGE×COND + GT×COND + SEX×AGE×GT + SEX×GT×COND + AGE×GT×COND	2.99e×10-3	0	0	0	55.65
SEX + AGE + GT + COND + SEX×GT + AGE×GT + SEX×COND + GT×COND	2.99e×10-3	0	0	0	14.14
SEX + AGE + GT + COND	2.99e×10-3	0	0	0	13.73
SEX + AGE + GT + COND + SEX×AGE + SEX×GT + AGE×GT + SEX×COND + AGE×COND + GT×COND + SEX×AGE×COND	2.99e×10-3	0	0	0	13.99
SEX + AGE + GT + COND + SEX×AGE + SEX×GT + AGE×GT + AGE×COND + GT×COND + AGE×GT×COND	2.99e×10-3	0	0	0	14.93
SEX + AGE + GT + COND + SEX×AGE + SEX×GT + AGE×GT + SEX×COND + AGE×COND + GT×COND + SEX×AGE×COND + AGE×GT×COND	2.99e×10-3	0	0	0	17.2
SEX + GT + COND + GT×COND	2.99e×10-3	0	0	0	13.6
SEX + AGE + GT + COND + AGE×GT + AGE×COND + GT×COND	2.99e×10-3	0	0	0	50.87
SEX + AGE + GT + COND + SEX×AGE + SEX×GT + AGE×COND	2.99e×10-3	0	0	0	13.86
SEX	2.99e×10-3	0	0	0	13.43
SEX + AGE + GT + COND + SEX×GT + AGE×GT + AGE×COND	2.99e×10-3	0	0	0	14.29
SEX + AGE + GT + COND + AGE×COND	2.99e×10-3	0	0	0	21.74
SEX + AGE + GT + COND + SEX×GT + SEX×COND	2.99e×10-3	0	0	0	14.8
SEX + AGE + GT + COND + SEX×COND + GT×COND	2.99e×10-3	0	0	0	15.61
SEX + AGE + GT + SEX×AGE + SEX×GT + AGE×GT	2.99e×10-3	0	0	0	13.91
SEX + AGE + GT + COND + SEX×AGE + SEX×GT + SEX×COND + AGE×COND + SEX×AGE×COND	2.99e×10-3	0	0	0	14.05
SEX + AGE + GT + COND + SEX×AGE + SEX×COND + AGE×COND + GT×COND + SEX×AGE×COND	2.99e×10-3	0	0	0	16.62
SEX + AGE + GT + COND + SEX×AGE + SEX×GT + AGE×GT + SEX×COND + AGE×COND + GT×COND + SEX×AGE×GT + SEX×AGE×COND	2.99e×10-3	0	0	0	26.63
SEX + AGE + GT + COND + SEX×AGE + SEX×GT + SEX×COND + AGE×COND + GT×COND	2.99e×10-3	0	0	0	14.12

SEX + AGE + GT + COND + SEX×AGE + SEX×GT + AGE×GT + SEX×COND + AGE×COND + GT×COND + SEX×AGE×COND + SEX×GT×COND	2.99e×10-3	0	0	0	21.88
SEX + AGE + GT + COND + SEX×AGE + SEX×GT + AGE×GT + AGE×COND + GT×COND + SEX×AGE×GT	2.99e×10-3	0	0	0	14.99
SEX + GT + COND + SEX×GT + SEX×COND + GT×COND	2.99e×10-3	0	0	0	13.78
SEX + AGE + GT + COND + SEX×GT + AGE×GT + SEX×COND + AGE×COND + GT×COND	2.99e×10-3	0	0	0	34.55
SEX + AGE + GT + COND + SEX×AGE + AGE×COND + GT×COND	2.99e×10-3	0	0	0	13.98
SEX + AGE + GT + COND + SEX×AGE + SEX×GT + SEX×COND + GT×COND + SEX×GT×COND	2.99e×10-3	0	0	0	17.88
SEX + AGE + GT + COND + SEX×AGE + SEX×GT + AGE×GT + SEX×COND + AGE×COND + GT×COND + SEX×AGE×COND + SEX×GT×COND + AGE×GT×COND	2.99e×10-3	0	0	0	16.41
SEX + AGE + GT + COND + SEX×GT + AGE×GT + SEX×COND + GT×COND + SEX×GT×COND	2.99e×10-3	0	0	0	14.07
SEX + AGE + GT + COND + SEX×COND + AGE×COND + GT×COND	2.99e×10-3	0	0	0	13.89
SEX + AGE + GT + COND + SEX×GT + SEX×COND + AGE×COND	2.99e×10-3	0	0	0	15.12
SEX + AGE + GT + COND + SEX×GT + AGE×GT + SEX×COND + AGE×COND + GT×COND + AGE×GT×COND	2.99e×10-3	0	0	0	16.68
SEX + AGE + GT + COND + SEX×AGE + SEX×GT + AGE×GT + SEX×COND + AGE×COND + GT×COND + SEX×AGE×GT + SEX×AGE×COND + AGE×GT×COND	2.99e×10-3	0	0	0	17.54
SEX + AGE + GT + COND + AGE×GT + AGE×COND + GT×COND + AGE×GT×COND	2.99e×10-3	0	0	0	14
SEX + AGE + GT + COND + SEX×AGE + SEX×GT + GT×COND	2.99e×10-3	0	0	0	14.76
SEX + AGE + GT + COND + SEX×AGE + SEX×GT + AGE×GT + SEX×COND + AGE×COND + GT×COND + SEX×AGE×GT + SEX×AGE×COND + SEX×GT×COND	2.99e×10-3	0	0	0	47.24
SEX + AGE + GT + COND + SEX×GT	2.99e×10-3	0	0	0	14.02
SEX + AGE + GT + SEX×AGE + SEX×GT + AGE×GT + SEX×AGE×GT	2.99e×10-3	0	0	0	14.72
SEX + AGE + GT + COND + SEX×AGE + SEX×GT + AGE×GT + AGE×COND + GT×COND + SEX×AGE×GT + AGE×GT×COND	2.99e×10-3	0	0	0	14.71
SEX + AGE + SEX×AGE	2.99e×10-3	0	0	0	13.65
SEX + AGE + GT + AGE×GT	2.99e×10-3	0	0	0	14.27
SEX + AGE + GT + COND + SEX×AGE + SEX×GT + SEX×COND + AGE×COND + GT×COND + SEX×GT×COND	2.99e×10-3	0	0	0	31.92
SEX + AGE + GT + COND + GT×COND	2.99e×10-3	0	0	0	26.8
SEX + GT + COND + SEX×GT + GT×COND	2.99e×10-3	0	0	0	13.83
SEX + GT	2.99e×10-3	0	0	0	13.46
SEX + AGE + GT + COND + SEX×GT + AGE×COND	2.99e×10-3	0	0	0	13.73

SEX + AGE + GT + COND + SEX×GT + AGE×GT + SEX×COND + AGE×COND + GT×COND + SEX×GT×COND	2.99e×10-3	0	0	0	15.35
SEX + AGE + GT + COND + SEX×GT + AGE×GT + AGE×COND + GT×COND	2.99e×10-3	0	0	0	15.53
SEX + AGE + GT + COND + SEX×AGE + SEX×GT + AGE×COND + GT×COND	2.99e×10-3	0	0	0	15.23
SEX + AGE + GT + COND + SEX×GT + AGE×GT + SEX×COND + AGE×COND + GT×COND + SEX×GT×COND + AGE×GT×COND	2.99e×10-3	0	0	0	14.52
SEX + AGE + GT + COND + SEX×AGE + SEX×GT + SEX×COND + AGE×COND + GT×COND + SEX×AGE×COND	2.99e×10-3	0	0	0	14.62
SEX + GT + COND + SEX×GT + SEX×COND + GT×COND + SEX×GT×COND	2.99e×10-3	0	0	0	13.95
SEX + AGE + GT + COND + SEX×GT + SEX×COND + GT×COND	2.99e×10-3	0	0	0	15.93
SEX + AGE + GT + COND + SEX×AGE + SEX×GT + AGE×GT + SEX×COND + AGE×COND + GT×COND + SEX×AGE×GT + SEX×AGE×COND + SEX×GT×COND + AGE×GT×COND	2.99e×10-3	0	0	0	20.28
SEX + AGE	2.99e×10-3	0	0	0	13.47
SEX + AGE + GT + COND + AGE×COND + GT×COND	2.99e×10-3	0	0	0	13.76
SEX + AGE + GT + COND + SEX×GT + AGE×GT + AGE×COND + GT×COND + AGE×GT×COND	2.99e×10-3	0	0	0	14.35
SEX + AGE + GT + SEX×AGE	2.99e×10-3	0	0	0	13.55
SEX + AGE + GT + SEX×GT + AGE×GT	2.99e×10-3	0	0	0	13.67
SEX + AGE + GT + COND + SEX×GT + SEX×COND + AGE×COND + GT×COND	2.99e×10-3	0	0	0	17.17
SEX + GT + SEX×GT	2.99e×10-3	0	0	0	13.73
SEX + AGE + GT + COND + SEX×AGE + SEX×GT + AGE×GT + SEX×COND + AGE×COND + GT×COND + SEX×AGE×GT + SEX×AGE×COND + SEX×GT×COND + AGE×GT×COND + SEX×AGE×GT×COND	2.99e×10-3	0	0	0	15.91
SEX + AGE + GT + COND + SEX×GT + GT×COND	2.99e×10-3	0	0	0	13.75
SEX + AGE + GT + COND + SEX×AGE + SEX×GT + SEX×COND + AGE×COND + GT×COND + SEX×AGE×COND + SEX×GT×COND	2.99e×10-3	0	0	0	23.94
SEX + AGE + GT + SEX×AGE + SEX×GT	2.99e×10-3	0	0	0	13.64
SEX + AGE + GT + COND + SEX×GT + SEX×COND + GT×COND + SEX×GT×COND	2.99e×10-3	0	0	0	14.35
SEX + AGE + GT	2.99e×10-3	0	0	0	13.85
SEX + AGE + GT + COND + SEX×GT + AGE×COND + GT×COND	2.99e×10-3	0	0	0	13.98
SEX + AGE + GT + COND + SEX×GT + SEX×COND + AGE×COND + GT×COND + SEX×GT×COND	2.99e×10-3	0	0	0	14.63
SEX + AGE + GT + SEX×GT	2.99e×10-3	0	0	0	13.55
COND	2.99e×10-3	0	0	0	13.43

AGE + COND	2.99e×10-3	0	0	0	13.8
GT + COND	2.99e×10-3	0	0	0	13.49
GT + COND + GT×COND	2.99e×10-3	0	0	0	13.52
AGE + COND + AGE×COND	2.99e×10-3	0	0	0	13.58
AGE + GT + COND	2.99e×10-3	0	0	0	13.52
AGE + GT + COND + AGE×GT	2.99e×10-3	0	0	0	13.57
AGE + GT + COND + GT×COND	2.99e×10-3	0	0	0	13.69
AGE + GT + COND + AGE×GT + GT×COND	2.99e×10-3	0	0	0	13.96
AGE + GT + COND + AGE×COND	2.99e×10-3	0	0	0	13.66
AGE + GT + COND + AGE×GT + AGE×COND	2.99e×10-3	0	0	0	13.74
AGE + GT + COND + AGE×COND + GT×COND	2.99e×10-3	0	0	0	13.64
AGE + GT + COND + AGE×GT + AGE×COND + GT×COND	2.99e×10-3	0	0	0	14.62
AGE + GT + COND + AGE×GT + AGE×COND + GT×COND + AGE×GT×COND	2.99e×10-3	0	0	0	14.37
Null model	2.99e×10-3	0	0	0	13.43
GT	2.99e×10-3	0	0	0	13.43
AGE	2.99e×10-3	0	0	0	13.43
AGE + GT	2.99e×10-3	0	0	0	13.55
AGE + GT + AGE×GT	2.99e×10-3	0	0	0	15.07

GT – *E(z)/w* genotype, COND – combination of experimental conditions (maintaining in the dark, low ambient temperature, a combination of rapamycin, berberine, and fucoxanthin, and dietary restriction), P(M) – prior model probability, P(M|data) – posterior model probabilities, BF_M – change from prior odds to posterior odds for each model, BF_{10} – Bayes factor. The most probable models are in bold.

Supplementary Table 15. Three-way Bayesian ANCOVA for the retrotransposon activity in males and females factored by age, genotype, and experimental conditions

Models	P(M)	P(M|data)	B_M	B_{10}	error %
Males					
**AGE + GT + COND + ANCOVA + AGE×GT + AGE×COND + GT×COND + AGE×GT×COND**	**0.03**	**1**	**800069.83**	**1**	
AGE + GT + COND + ANCOVA + AGE×GT + GT×COND	0.03	3.23e×10-5	1.19e×10-3	3.23e×10-5	6.47
AGE + GT + COND + ANCOVA + AGE×GT + AGE×COND + GT×COND	0.03	1.19e×10-5	4.40e×10-4	1.19e×10-5	6.12
AGE + GT + COND + ANCOVA + AGE×GT	0.03	1.75e×10-6	6.49e×10-5	1.75e×10-6	5.49
AGE + GT + COND + ANCOVA + AGE×GT + AGE×COND	0.03	3.21e×10-7	1.19e×10-5	3.21e×10-7	6.69
AGE + GT + COND + ANCOVA + AGE×COND + GT×COND	0.03	6.64e×10-19	2.46e×10-17	6.64e×10-19	5.92
AGE + GT + COND + ANCOVA + AGE×COND	0.03	3.07e×10-19	1.14e×10-17	3.07e×10-19	5.53
AGE + COND + ANCOVA + AGE×COND	0.03	2.11e×10-19	7.79e×10-18	2.11e×10-19	5.81
AGE + GT + COND + ANCOVA	0.03	3.98e×10-20	1.47e×10-18	3.98e×10-20	5.29
AGE + GT + COND + ANCOVA + GT×COND	0.03	3.45e×10-20	1.28e×10-18	3.45e×10-20	5.62
AGE + COND + ANCOVA	0.03	3.03e×10-20	1.12e×10-18	3.03e×10-20	5.13
AGE + GT + ANCOVA + AGE×GT	0.03	9.03e×10-28	3.34e×10-26	9.03e×10-28	5.56
COND + ANCOVA	0.03	3.68e×10-29	1.36e×10-27	3.68e×10-29	4.87
GT + COND + ANCOVA	0.03	7.82e×10-30	2.89e×10-28	7.82e×10-30	5.21
GT + COND + ANCOVA + GT×COND	0.03	5.83e×10-30	2.16e×10-28	5.83e×10-30	5.8
AGE + GT + ANCOVA	0.03	1.16e×10-39	4.27e×10-38	1.16e×10-39	5.87
AGE + ANCOVA	0.03	3.65e×10-43	1.35e×10-41	3.65e×10-43	5.09
GT + ANCOVA	0.03	5.00e×10-45	1.85e×10-43	5.00e×10-45	4.93
ANCOVA	0.03	8.87e×10-47	3.28e×10-45	8.87e×10-47	4.7
AGE + GT + COND + AGE×GT	0.03	4.26e×10-268	1.58e×10-266	4.26e×10-268	14.63
AGE + GT + COND + AGE×GT + GT×COND	0.03	1.16e×10-268	4.28e×10-267	1.16e×10-268	5.14
AGE + COND	0.03	6.39e×10-269	2.37e×10-267	6.39e×10-269	4.86

AGE + GT + COND + AGE×GT + AGE×COND	0.03	4.83e×10-269	1.79e×10-267	4.83e×10-269	5.44
AGE + GT + COND + AGE×GT + AGE×COND + GT×COND + AGE×GT×COND	0.03	2.47e×10-269	9.12e×10-268	2.47e×10-269	6.89
AGE + COND + AGE×COND	0.03	1.72e×10-269	6.36e×10-268	1.72e×10-269	6.38
AGE + GT + COND + AGE×GT + AGE×COND + GT×COND	0.03	1.60e×10-269	5.91e×10-268	1.60e×10-269	6.35
AGE + GT + COND	0.03	1.08e×10-269	4.00e×10-268	1.08e×10-269	5.35
COND	0.03	5.33e×10-270	1.97e×10-268	5.33e×10-270	4.7
AGE + GT + COND + AGE×COND	0.03	2.76e×10-270	1.02e×10-268	2.76e×10-270	5.5
AGE + GT + COND + GT×COND	0.03	1.92e×10-270	7.10e×10-269	1.92e×10-270	5.96
AGE + GT + COND + AGE×COND + GT×COND	0.03	5.98e×10-271	2.21e×10-269	5.98e×10-271	5.43
GT + COND	0.03	5.55e×10-271	2.05e×10-269	5.55e×10-271	4.99
GT + COND + GT×COND	0.03	1.08e×10-271	4.01e×10-270	1.09e×10-271	6.21
AGE + GT + AGE×GT	0.03	5.00e×10-272	1.85e×10-270	5.00e×10-272	4.92
AGE + GT	0.03	1.63e×10-273	6.04e×10-272	1.63e×10-273	4.84
AGE	0.03	1.41e×10-273	5.22e×10-272	1.41e×10-273	4.7
Null odel	0.03	1.30e×10-273	4.81e×10-272	1.30e×10-273	4.7
GT	0.03	6.22e×10-274	2.30e×10-272	6.22e×10-274	4.7
Females					
**AGE + GT + ANCOVA + AGE×GT**	**0.03**	**0.48**	**33.7**	**1**	
AGE + ANCOVA	0.03	0.19	8.93	0.41	6.73
AGE + GT + COND + ANCOVA + AGE×GT	0.03	0.11	4.72	0.24	7.47
AGE + COND + ANCOVA	0.03	0.05	2.01	0.11	6.98
AGE + GT + COND + ANCOVA + AGE×GT + AGE×COND	0.03	0.05	1.8	0.1	10.62
AGE + COND + ANCOVA + AGE×COND	0.03	0.04	1.36	0.07	7.2
AGE + GT + COND + ANCOVA + AGE×GT + GT×COND	0.03	0.03	0.98	0.05	8.11
AGE + GT + ANCOVA	0.03	0.02	0.71	0.04	6.96
AGE + GT + COND + ANCOVA + AGE×GT + AGE×COND + GT×COND	0.03	0.01	0.42	0.02	14.33
ANCOVA	0.03	9.64e×10-3	0.36	0.02	6.54

AGE + GT + COND + ANCOVA	0.03	4.95e×10-3	0.18	0.01	7.78
AGE + GT + COND + ANCOVA + AGE×COND	0.03	3.63e×10-3	0.13	7.62e×10-3	8.04
AGE + GT + COND + ANCOVA + AGE×GT + AGE×COND + GT×COND + AGE×GT×COND	0.03	2.72e×10-3	0.1	5.70e×10-3	10.19
COND + ANCOVA	0.03	2.45e×10-3	0.09	5.14e×10-3	6.83
AGE + GT + COND + ANCOVA + GT×COND	0.03	1.17e×10-3	0.04	2.44e×10-3	7.67
GT + ANCOVA	0.03	9.32e×10-4	0.03	1.95e×10-3	6.74
AGE + GT + COND + ANCOVA + AGE×COND + GT×COND	0.03	8.35e×10-4	0.03	1.75e×10-3	16.03
GT + COND + ANCOVA	0.03	2.43e×10-4	8.99e×10-3	5.10e×10-4	7.1
GT + COND + ANCOVA + GT×COND	0.03	5.52e×10-5	2.04e×10-3	1.16e×10-4	7.24
Null odel	0.03	1.36e×10-199	5.04e×10-198	2.86e×10-199	6.54
AGE	0.03	4.04e×10-200	1.49e×10-198	8.48e×10-200	6.54
COND	0.03	1.59e×10-200	5.89e×10-199	3.34e×10-200	6.54
GT	0.03	1.26e×10-200	4.68e×10-199	2.65e×10-200	6.54
AGE + COND	0.03	4.59e×10-201	1.70e×10-199	9.63e×10-201	6.69
AGE + GT	0.03	3.52e×10-201	1.30e×10-199	7.39e×10-201	6.73
GT + COND	0.03	1.40e×10-201	5.20e×10-200	2.95e×10-201	6.67
AGE + GT + AGE×GT	0.03	1.29e×10-201	4.79e×10-200	2.71e×10-201	7.29
AGE + COND + AGE×COND	0.03	8.19e×10-202	3.03e×10-200	1.72e×10-201	6.73
AGE + GT + COND	0.03	4.36e×10-202	1.61e×10-200	9.16e×10-202	7.64
GT + COND + GT×COND	0.03	2.17e×10-202	8.03e×10-201	4.55e×10-202	8.85
AGE + GT + COND + AGE×GT	0.03	1.55e×10-202	5.72e×10-201	3.24e×10-202	7.37
AGE + GT + COND + AGE×COND	0.03	7.63e×10-203	2.82e×10-201	1.60e×10-202	7.59
AGE + GT + COND + GT×COND	0.03	5.73e×10-203	2.12e×10-201	1.20e×10-202	7.12
AGE + GT + COND + AGE×GT + AGE×COND	0.03	2.73e×10-203	1.01e×10-201	5.73e×10-203	7.1
AGE + GT + COND + AGE×GT + GT×COND	0.03	2.11e×10-203	7.80e×10-202	4.42e×10-203	7.93
AGE + GT + COND + AGE×COND + GT×COND	0.03	1.03e×10-203	3.80e×10-202	2.15e×10-203	7.69
AGE + GT + COND + AGE×GT + AGE×COND + GT×COND	0.03	8.17e×10-204	3.02e×10-202	1.71e×10-203	61.47

AGE + GT + COND + AGE×GT + AGE×COND + GT×COND + AGE×GT×COND	0.03	6.72e×10-205	2.48e×10-203	1.41e×10-204	7.5

GT – *E(z)/w* genotype, COND – combination of experimental conditions (maintaining in the dark, low ambient temperature, a combination of rapamycin, berberine, and fucoxanthin, and dietary restriction), P(M) – prior model probability, P(M|data) – posterior model probabilities, BF_M – change from prior odds to posterior odds for each model, BF_{10} – Bayes factor. The most probable models are in bold.

Supplementary Table 16. The list of experimental groups for transcriptome analysis

Variant	Sex	Replicate	Age (days)			
			5	50	100	150
w/w (control)	♂	1	M1w5C	M1w50C	n/a	n/a
w/w (DR, 3G, 18°C, DD)	♂	1	M1w5E	M1w50E	M1w100E	M1w150E
E(z)/w (control)	♂	1	M1Ez5C	M1Ez50C	n/a	n/a
E(z)/w (DR, 3G, 18°C, DD)	♂	1	M1Ez5E	M1Ez50E	M1Ez100E	M1Ez150E
w/w (control)	♂	2	M2w5C	M2w50C	n/a	n/a
w/w (DR, 3G, 18°C, DD)	♂	2	M2w5E	M2w50E	M2w100E	M2w150E
E(z)/w (control)	♂	2	M2Ez5C	M2Ez50C	n/a	n/a
E(z)/w (DR, 3G, 18°C, DD)	♂	2	M2Ez5E	M2Ez50E	M2Ez100E	M2Ez150E
w/w (control)	♂	3	M3w5C	M3w50C	n/a	n/a
w/w (DR, 3G, 18°C, DD)	♂	3	M3w5E	M3w50E	M3w100E	M3w150E
E(z)/w (control)	♂	3	M3Ez5C	M3Ez50C	n/a	n/a
E(z)/w (DR, 3G, 18°C, DD)	♂	3	M3Ez5E	M3Ez50E	M3Ez100E	M3Ez150E
w/w (control)	♀	1	F1w5C	F1w50C	n/a	n/a
w/w (DR, 3G, 18°C, DD)	♀	1	F1w5E	F1w50E	F1w100E	F1w150E
E(z)/w (control)	♀	1	F1Ez5C	F1Ez50C	n/a	n/a
E(z)/w (DR, 3G, 18°C, DD)	♀	1	F1Ez5E	F1Ez50E	F1Ez100E	F1Ez150E
w/w (control)	♀	2	F2w5C	F2w50C	n/a	n/a
w/w (DR, 3G, 18°C, DD)	♀	2	F2w5E	F2w50E	F2w100E	n/a
E(z)/w (control)	♀	2	F2Ez5C	F2Ez50C	n/a	n/a
E(z)/w (DR, 3G, 18°C, DD)	♀	2	F2Ez5E	F2Ez50E	F2Ez100E	F2Ez150E
w/w (control)	♀	3	F3w5C	F3w50C	n/a	n/a
w/w (DR, 3G, 18°C, DD)	♀	3	F3w5E	F3w50E	F3w100E	n/a
E(z)/w (control)	♀	3	F3Ez5C	F3Ez50C	n/a	n/a
E(z)/w (DR, 3G, 18°C, DD)	♀	3	F3Ez5E	F3Ez50E	F3Ez100E	F3Ez150E

Supplementary Table 17. List of primers for qRT-PCR

Gene	Forward (5'-3')	Reverse (5'-3')
Het-A1	cgcgcggaacccatcttcaga	cgccgcagtcgtttggtgagt
R1-element	cgttgtttccactgccgtta	cctaggctgcggaaactgat
Rt1a	ccacacagactgaggcagaa	acgcataactttccggtttg
1731	agcaaacgtctgttggaagg	cgacagcaaaacaacactgc
412	caccggtttggtcgaaag	ggacatgcctggtattttgg
blood	tgccacagtacctgatttcg	gattcgccttttacgtttgc
opus	cgaggagtggggagagattg	tgcgaaaatctgcctgaacc
roo	cgtctgcaatgtactggctct	cggcactccactaacttctcc
LINE-1	ggccatgtccgtctgtcc	agctagtgtgaatgcgaacg
eukaryotic translation elongation factor 1 alpha 2	agggcaagaagtagctggtttgc	gctgctactactgcgtgttgttg
β-Tubulin at 56D	gcaactccactgccatcc	cctgctcctcctcgaact
Ribosomal protein L32	gaagcgcaccaagcacttcatc	cgccatttgtgcgacagcttag
