# Supplementary material for: Molecular mechanisms of exceptional lifespan increase of Drosophila melanogaster with different genotypes after combinations of pro-longevity interventions
Source: Commun Biol. 2022 Jun 9;5:566. doi: 10.1038/s42003-022-03524-4 (PMC9184560; doi:10.1038/s42003-022-03524-4)
Supplement: Supplementary file 3 — Description of Additional Supplementary Files [file 42003_2022_3524_MOESM3_ESM.pdf]

Description of Additional Supplementary Files

File name: Supplementary Data 1
Description: Complete lists of differentially expressed genes.

File name: Supplementary Data 2
Description: All source data underlying the graphs and charts presented in the main figures.
