# Supplementary material for: Molecular mechanisms of exceptional lifespan increase of Drosophila melanogaster with different genotypes after combinations of pro-longevity interventions
Source: Commun Biol. 2022 Jun 9;5:566. doi: 10.1038/s42003-022-03524-4 (PMC9184560; doi:10.1038/s42003-022-03524-4)
Supplement: Supplementary file 6 — Reporting Summary [file 42003_2022_3524_MOESM6_ESM.pdf]

nature portfolio

Corresponding author(s): Alexey A. Moskalev

Last updated by author(s): May 23, 2022

Reporting Summary

Nature Portfolio wishes to improve the reproducibility of the work that we publish. This form provides structure for consistency and transparency in reporting. For further information on Nature Portfolio policies, see our Editorial Policies and the Editorial Policy Checklist.

Statistics

For all statistical analyses, confirm that the following items are present in the figure legend, table legend, main text, or Methods section.

n/a	Confirmed	
☐	☒	The exact sample size (*n*) for each experimental group/condition, given as a discrete number and unit of measurement
☐	☒	A statement on whether measurements were taken from distinct samples or whether the same sample was measured repeatedly
☐	☒	The statistical test(s) used AND whether they are one- or two-sided<br>*Only common tests should be described solely by name; describe more complex techniques in the Methods section.*
☐	☒	A description of all covariates tested
☐	☒	A description of any assumptions or corrections, such as tests of normality and adjustment for multiple comparisons
☐	☒	A full description of the statistical parameters including central tendency (e.g. means) or other basic estimates (e.g. regression coefficient) AND variation (e.g. standard deviation) or associated estimates of uncertainty (e.g. confidence intervals)
☐	☒	For null hypothesis testing, the test statistic (e.g. *F*, *t*, *r*) with confidence intervals, effect sizes, degrees of freedom and *P* value noted<br>*Give P values as exact values whenever suitable.*
☐	☒	For Bayesian analysis, information on the choice of priors and Markov chain Monte Carlo settings
☒	☐	For hierarchical and complex designs, identification of the appropriate level for tests and full reporting of outcomes
☒	☐	Estimates of effect sizes (e.g. Cohen's *d*, Pearson's *r*), indicating how they were calculated

Our web collection on statistics for biologists contains articles on many of the points above.

Software and code

Policy information about availability of computer code

Data collection	The locomotor activity data were collected using the Locomotor Activity Monitors (TriKinetics, USA) and DAMSystem311 (TriKinetics, USA) data acquisition software. The RNA-Seq was performed on the NextSeq 500 System (Illumina, USA).
Data analysis	We have described statistical analyses in the manuscript. The statistical analyses of lifespan, lipid content, stress resistance, and locomotor activity data were conducted by using Statistica Ultimate Academic (version 13.3, StatSoft, USA), OASIS 2 (Online application for survival analysis), JASP (version 0.16, JASP team, Netherlands), and Microsoft Excel 2019 (Microsoft, USA). Statistical data processing of qRT-PCR was performed using the CFX96 Software (BioRad, USA). The analysis of RNA-Seq data was performed using Trimmomatic 0.39, FastQC 0.11.9, STAR 2.7, RSeQC 3.0.1, Subread 1.6.0, as well as R Bioconductor packages: edgeR 3.28.1, topGO 2.38, clusterProfiler 3.14.3, pathview 1.12.

For manuscripts utilizing custom algorithms or software that are central to the research but not yet described in published literature, software must be made available to editors and reviewers. We strongly encourage code deposition in a community repository (e.g. GitHub). See the Nature Portfolio guidelines for submitting code & software for further information.

Data

Policy information about availability of data

All manuscripts must include a data availability statement. This statement should provide the following information, where applicable:

- Accession codes, unique identifiers, or web links for publicly available datasets
- A description of any restrictions on data availability
- For clinical datasets or third party data, please ensure that the statement adheres to our policy

The sequencing data are available at the NCBI Sequence Read Archive (PRJNA757594).

All source data underlying the graphs and charts presented in the main figures are available in Supplementary Data 1.
The authors declare that all other data supporting the findings of this study are available within the article and its supplementary information files.

Field-specific reporting

Please select the one below that is the best fit for your research. If you are not sure, read the appropriate sections before making your selection.

☒ Life sciences ☐ Behavioural & social sciences ☐ Ecological, evolutionary & environmental sciences

For a reference copy of the document with all sections, see nature.com/documents/nr-reporting-summary-flat.pdf

Life sciences study design

All studies must disclose on these points even when the disclosure is negative.

Sample size	Sample sizes were determined based on previous experience and the literature. No statistical methods were used to predetermine sample sizes.
Data exclusions	No data were excluded from the analyses.
Replication	All experiments were performed at least twice. The most of experiments were performed in 3 (usually) - 12 (in some cases) independent biological replicates. RNA-Seq analysis was performed in triplicates.
Randomization	The flies were randomly allocated into experimental groups.
Blinding	The investigator were blinded to group allocation during data collection by using numeric codes.

Reporting for specific materials, systems and methods

We require information from authors about some types of materials, experimental systems and methods used in many studies. Here, indicate whether each material, system or method listed is relevant to your study. If you are not sure if a list item applies to your research, read the appropriate section before selecting a response.

Materials & experimental systems

n/a	Involved in the study	
☒	☐	Antibodies
☒	☐	Eukaryotic cell lines
☒	☐	Palaeontology and archaeology
☐	☒	Animals and other organisms
☒	☐	Human research participants
☒	☐	Clinical data
☒	☐	Dual use research of concern

Methods

n/a	Involved in the study	
☒	☐	ChIP-seq
☒	☐	Flow cytometry
☒	☐	MRI-based neuroimaging

Animals and other organisms

Policy information about studies involving animals; ARRIVE guidelines recommended for reporting animal research

Laboratory animals	The Drosophila melanogaster lines were used which were purchased from the Bloomington Drosophila Stock Centre (Bloomington, USA): E(z)731/TM6C (#24470, Bloomington, USA) and w1118 (#3605, Bloomington, USA).
Wild animals	The study did not involve wild animals.
Field-collected samples	The study did not involve samples collected from the field.
Ethics oversight	This study involves the use of model organism Drosophila melanogaster, which does not need any ethical approval.

Note that full information on the approval of the study protocol must also be provided in the manuscript.
